# Investigation on *N*-Aryl-2-(4-sulfamoylphenyl)hydrazine-1-carbothioamide as Human Carbonic Anhydrases Inhibitors

**DOI:** 10.3390/ph19010151

**Published:** 2026-01-14

**Authors:** Morteza Abdoli, Andrea Angeli, Alessandro Bonardi, Paola Gratteri, Ludmila Jackevica, Antons Sizovs, Claudiu T. Supuran, Raivis Žalubovskis

**Affiliations:** 1 Latvian Institute of Organic Synthesis, LV-1006 Riga, Latvia; 2 Department of Neurofarba, Università degli Studi di Firenze, 50019 Florence, Italy; 3 Pharmaceutical and Nutraceutical Section and Laboratory of Molecular Modeling Cheminformatics & QSAR, Department of NEUROFARBA, University of Florence, Via U. Schiff 6, Sesto Fiorentino, 50019 Florence, Italy; 4 Baltic Biomaterials Centre of Excellence, Headquarters at Riga Technical University, LV-1048 Riga, Latvia; 5 Institute of Chemistry and Chemical Technology, Faculty of Natural Sciences and Technology, Riga Technical University, LV-1048 Riga, Latvia

**Keywords:** carbonic anhydrase, cancer-associated CA isoforms, transmembrane isoforms, inhibitors, sulfonamides, *N*-aryl-2-(4-sulfamoylphenyl)hydrazine-1-carbothioamide

## Abstract

**Background**: Among the 15 human (h) carbonic anhydrase (CA; EC 4.2.1.1) isoforms, hCA IX and XII are particularly important due to their roles in tumor cell growth and survival, identifying them as promising targets for anticancer therapy. As a result, considerable effort has been directed toward the development of novel inhibitors that are highly selective for these isoforms. **Methods**: A library of twelve novel *N*-aryl-2-(4-sulfamoylphenyl)hydrazine-1-carbothioamides **3** along with two new *N*-aryl-2-(4-sulfamoylphenyl)hydrazine-1-carboxamide derivatives **5** were synthesized and their inhibition abilities were tested against four human carbonic anhydrase isozymes (hCA I, II, IX and XII) related to some global diseases including glaucoma, cancer and osteoporosis. **Results**: All compounds exhibited potent inhibition of the tested isoforms in the nanomolar range. Compound **3i** showed the highest inhibition of hCA I activity but demonstrated poor selectivity toward the other isoforms. Compound **3h** displayed superior selectivity for hCA II over hCA I (hCA I/II = 37) and exhibited 2.5-fold higher inhibitory activity compared to acetazolamide (AAZ). Among the tested compounds, **3l** (*K*_i_ = 32.1 nM) demonstrated markedly improved selectivity for hCA IX over hCA I, II, and XII relative to the standard drug. Notably, compound **3a** showed the most potent inhibition against hCA XII (*K*_i_ = 6.8 nM), comparable to AAZ, while exhibiting significantly greater selectivity over off-target isoforms and the other tumor-associated isozyme (hCA IX/XII = 20 versus hCA IX/XII = 4.5 for AAZ). **Conclusions**: The present study suggests potent lead compounds as selective hCA IX and XII inhibitors with anticancer activity.

## 1. Introduction

Carbonic anhydrases (CAs, EC 4.2.1.1) comprise a family of lyase enzymes that are widely distributed across living organisms [1]. To date, 15 isoforms of CA have been discovered in humans and among them, 12 isoforms (CA I–IV, VA–VB, VI–VII, IX, and XII–XIV) possess zinc in their active site and exhibit catalytic activity [2]. The catalytic activity of these isoforms is identical (facilitating the reversible hydration of carbon dioxide and water into protons and bicarbonate); however, they differ in oligomeric arrangement, cellular localization, tissue distribution, expression levels, and kinetic properties [3]. The CA-catalyzed reaction is involved in numerous physiological processes across various tissues and cellular compartments, including respiration, pH regulation, electrolyte transport, and metabolism [4]. Abnormal levels or activity of CAs can lead to various pathological conditions related to pH buffering, metabolism, signaling, and other processes [5]. Therefore, the selective inhibition of specific CA isoforms is considered as a promising therapeutic approach for diseases resulting from their abnormal activity [6]. In this context, the crucial role of plasma membrane-associated CA isoforms, CA IX and CA XII, in tumor progression, acidification, and metastasis has positioned them as significant therapeutic targets for cancer [7,8].

**Figure 1:**
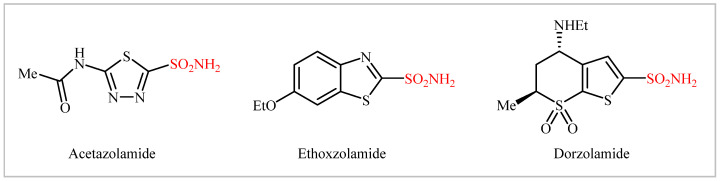
Examples of FDA-approved sulfonamide-based CAIs.

Among the various pharmacophores reported as Zn-binding groups that can fit/block the active site of CAs, sulfonamides are the most effective group in both marketed and reported CA inhibitors (CAIs) (Figure 1) [9,10]. However, they are often associated with severe side effects due to their poor selectivity and potent binding to multiple CA isoforms [11]. Therefore, further structural modifications are necessary to develop more effective and truly selective CAIs.

**Figure 2:**
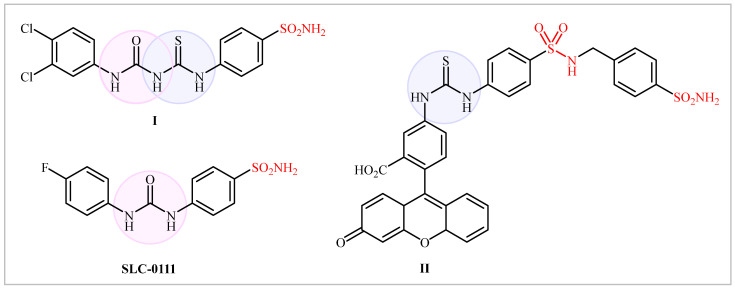
Examples of isoform selective sulfonamide-based CAIs having (thio)urea moieties.

Intriguingly, recent studies have shown that incorporating (thio)urea moieties into sulfonamide-containing compounds leads to CAIs with enhanced efficiency and selectivity [12,13,14,15]. For example (Figure 2), 4-(3-((3,4-dichlorophenyl)carbamoyl)thioureido)benzenesulfonamide **I** having acyl thiourea moiety has been demonstrated as an efficient in vitro inhibitor of *Plasmodium falciparum* CA (PfaCA) [16], while sulfonamide **II** which contains a thiourea moiety, has been identified as a potent inhibitor of CA IX [17]. More interestingly, SLC-0111, an unsymmetrical disubstituted urea derivative, is the only CA IX inhibitor that has successfully completed Phase I clinical trials [18,19,20].

**Figure 3:**
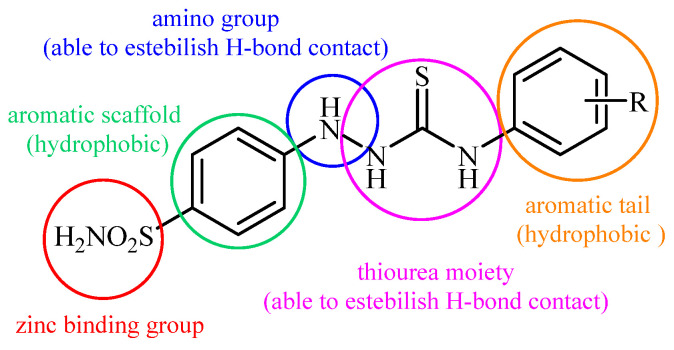
General structure of *N*-aryl-2-(4-sulfamoylphenyl)hydrazine-1-carbothioamides discussed in the paper.

Keeping these interesting findings in mind and continuing our research on the field of CAIs [21,22,23,24,25,26,27,28,29,30], herein, we aimed to design and synthesize a series of novel *N*-aryl-2-(4-sulfamoylphenyl)hydrazine-1-carbothioamides and evaluate their inhibitory activity against four human (h)CA isoforms, hCA I, II, IX and XII (Figure 3).

## 2. Results and Discussion

### 2.1. Synthesis

**Scheme 1:**
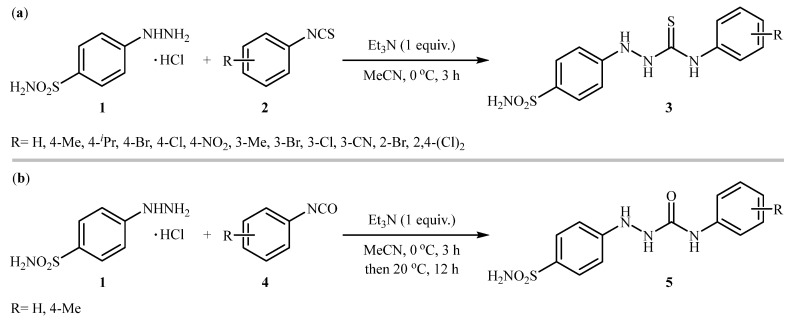
General synthetic scheme of (**a**) *N*-aryl-2-(4-sulfamoylphenyl)hydrazine-1-carbothioamides **3**; (**b**) *N*-aryl-2-(4-sulfamoylphenyl)hydrazine-1-carboxamides **5**.

A panel of twelve novel *N*-aryl-2-(4-sulfamoylphenyl)hydrazine-1-carbothioamide derivatives **3** were prepared by reaction of (4-sulfamoylphenyl)hydrazine hydrochloride **1** with equimolar quantities of respective aryl isothiocyanates **2** in acetonitrile under basic conditions (Scheme 1a). Two new *N*-aryl-2-(4-sulfamoylphenyl)hydrazine-1-carboxamides **5a** and **5b** were similarly synthesized by reaction of (4-sulfamoylphenyl)hydrazine hydrochloride **1** with aryl isocyanates **4** (Scheme 1b). The structures of the target compounds were confirmed by spectroscopic methods (^1^H NMR, ^13^C NMR and HRMS).

### 2.2. Carbonic Anhydrase Inhibition

All newly synthesized *N*-aryl-2-(4-sulfamoylphenyl)hydrazine-1-carbothioamides (**3a**–**3l**) were evaluated for their capacity to suppress four physiologically relevant hCA isoforms (hCA I, hCA II, hCA IX, and CA XII), which have been defined as drug targets. The evaluation was conducted using a stopped-flow CO_2_ hydrase assay [31], with acetazolamide (AAZ) serving as the standard inhibitor (Table 1).

According to the data in Table 1, the structure–activity relationships (SAR) of the titled compounds can be summarized as follows:

(i) The slow cytosolic isoform hCA I was effectively inhibited by all newly developed sulfonamides **3a**–**3l** with inhibition constants (*K*_i_) ranging from 7.7 nM to 0.7 µM, spanning low nanomolar to very low micromolar levels. A 91-fold variation across the series indicates that the inhibitory potency of this class of compounds is greatly influenced by the nature and position of substituents on the phenyl ring periphery of substrates. Overall, the relative inhibitory activity of the tested compounds followed the order: *meta*-substituted ≥ *para*-substituted > *ortho*-substituted derivatives. Regarding the nature of the substituents, a comparison of differently functionalized *para*-substituted derivatives revealed that compounds bearing a 4-nitro group (a hydroneutral moiety capable of forming hydrogen bonds) exhibited superior inhibitory potency compared to those with 4-alkyl substituents, which in turn outperformed the 4-halo-substituted analogs.
(ii) The physiologically relevant and ubiquitous cytosolic isoform hCA II was strongly inhibited by all investigated compounds of type **3** with *K*_i_ ranging from 4.3 to 78.8 nM compared to the standard AAZ with *K*_i_ value of 12.1 nM. Notably, two-thirds of these compounds exhibited superior inhibitory activity compared to the reference drug. Compound **3i** showed the best inhibition against hCA II with a *K*_i_ of 4.3 nM, which was almost 3-fold higher than that of AAZ. The SAR showed that *ortho*-substituted derivatives displayed weakest inhibitory capability against this isoform, similar to the trend observed for hCA I.
(iii) The tumor-associated transmembrane isoform hCA IX was inhibited strongly to moderately by all synthesized compounds **3a**–**3l**, with *K*_i_ values ranging from 23.9 to 251.0 nM, compared to standard AAZ (25.7 nM). The results showed that the nature of the substituents had a greater impact on the inhibitory activity against this isoform than their position on the aromatic ring. The five most potent compounds were **3e**, **3h**, **3i**, **3k** and **3l** with very low nanomolar *K*_i_ values 27.5, 31.2, 23.9, 68.7 and 32.1 nM, respectively. Interestingly, in these five molecules, R was a halide group (Cl or Br).
(iv) The other tumor-associated transmembrane isoform hCA XII was also effectively inhibited by all of the tested sulfonamides **3** with *K*_i_ values only differing by 4-fold and ranging between 5.5 and 23.1 nM. Here, no significant differences in inhibitory potency were observed among the molecules with different substituents. Compound **3f** displayed the best inhibition against this isoform, equal to that of AAZ, but significantly more selective over the other tumor-associated isoform hCA IX (hCA IX/XIII = 22) compared to AAZ (hCA IX/XIII = 4.5).

To compare the inhibitory potency of the newly developed compounds **3** with their urea analogues, two *N*-aryl-2-(4-sulfamoylphenyl)hydrazine-1-carboxamides **5** were synthesized and evaluated against the same set of isoforms (Table 2).

The results showed that both *N*-aryl-2-(4-sulfamoylphenyl)hydrazine-1-carboxamide derivatives **5a** and **5b** effectively inhibited all four tested isoforms, and they demonstrated potencies nearly equivalent to those of their corresponding carbothioamide analogues, **3a** and **3b**.

### 2.3. In Silico Studies

To investigate the binding mode of compound **3l**, the most selective of the series in respect of both target isoforms CA IX and CA XII compared to the cytosolic hCA I and II (hCA I/IX = 21.8; hCA II/IX = 2.5; hCA I/XII = 73.6; hCA II/XII = 8.3), combined docking and molecular dynamics (MD) simulations were performed.

As reported in the literature [32,33,34,35], all docking solutions placed the benzenesulfonamide moiety deeply within the active site, coordinating the zinc ion through the deprotonated sulfonamide nitrogen (SO_2_NH^−^) and completing the metal tetrahedral coordination sphere with H94, H96, and H119 in both isoforms. This coordination is further stabilized by two H-bonds between the sulfonamide NH^−^ and S=O groups and the side-chain OH and backbone NH of T199, respectively.

**Figure 4:**
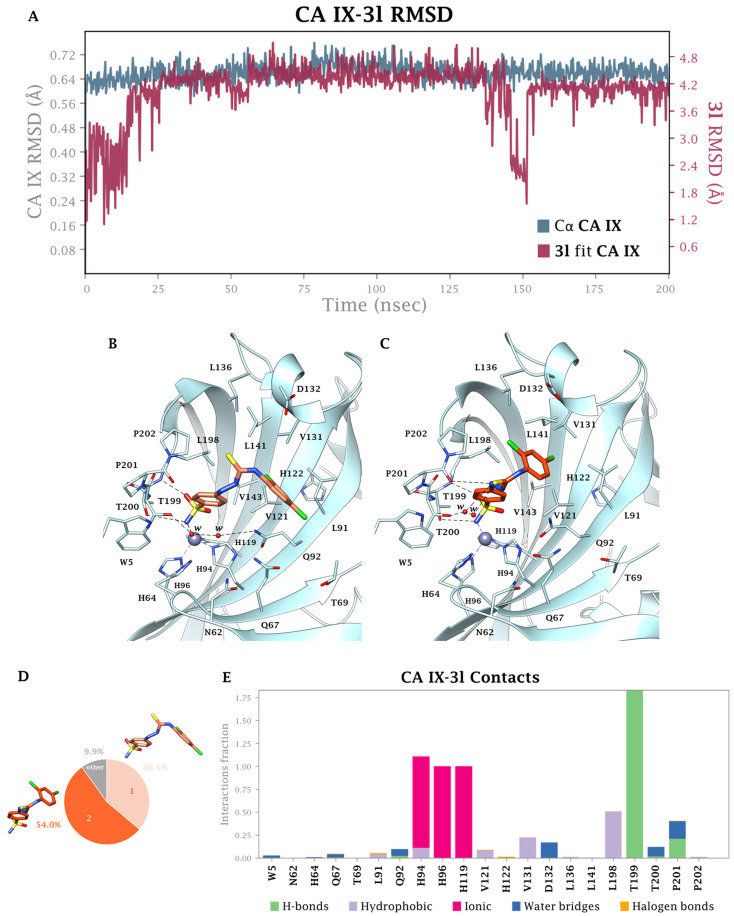
(**A**) 200 ns-long MD trajectory of the ligand **3l**–CA IX complex. (**B**,**C**) Representative binding modes of ligand **3l** for each cluster conformation. H-bonds are shown as black dashed lines. The ligand is depicted with progressively darker coloring along the course of the simulation. Water molecules and Zn^2+^ are represented as red and gray spheres, respectively. (**D**) Pie chart showing the representative conformers per cluster and their relative abundances. (**E**) Histogram of the contacts in the **3l**–CA IX complex. A value of 1.00 corresponds to 100% persistence, while values greater than 1.00 indicate that the same residue engages in more than one interaction with the ligand (e.g., T199 forms two H-bonds with the sulfonamide group of **3l**).

**Figure 5:**
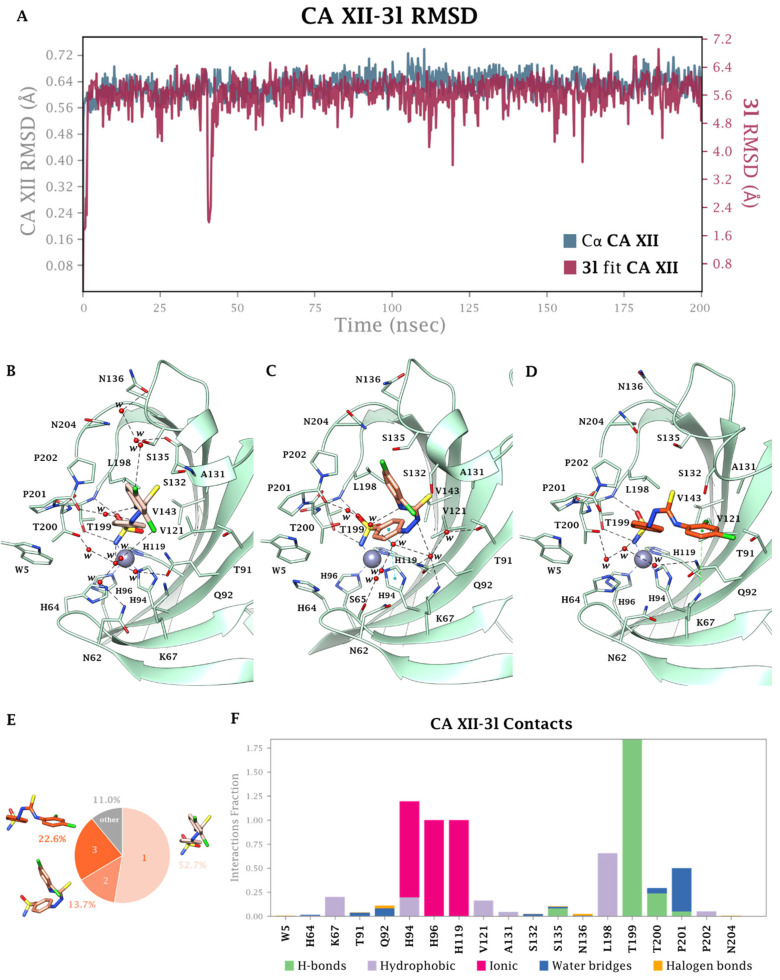
(**A**) 200 ns-long MD trajectory of the ligand **3l**–CA XII complex. (**B**–**D**) Representative binding modes of ligand **3l** for each cluster conformation. H-bonds, π–π stacking, and π–cation interactions are shown as black, cyan, and green dashed lines, respectively. The ligand is depicted with progressively darker coloring along the course of the simulation. Water molecules and Zn^2+^ are represented as red and gray spheres, respectively. (**E**) Pie chart showing the representative conformers per cluster and their relative abundances. (**F**) Histogram of the contacts in the **3l**–CA XII complex. A value of 1.00 corresponds to 100% persistence, while values greater than 1.00 indicate that the same residue engages in more than one interaction with the ligand (e.g., T199 forms two H-bonds with the sulfonamide group of **3l**).

The best score docking poses were subjected to 200 ns-long MD simulations to explore the time-dependent evolution of ligand–target interactions (Figure 4 and Figure 5).

In CA IX, ligand **3l** maintained an RMSD of ~4.4 Å for the first 130 ns, before fluctuating and stabilizing at ~3.9 Å for the rest of the simulation (Figure 4A). Two major binding mode clusters were identified (Figure 4B,C): the first persisted for 36.1% of the trajectory (Figure 4B,D), while the second was more abundant (54.0%) and representative (Figure 4C,D).

As shown in Figure 4E, the binding mode of the sulfonamide group remained stable throughout the 200-ns trajectory (100%), both in terms of Zn^2+^ coordination and hydrogen bonding with T199. In addition, hydrophobic van der Waals (vdW) interactions of the benzenesulfonamide ring with H94 (~11%), V121 (~7%), and L198 (~50%) contributed to stabilizing its orientation within the binding pocket.

In the first cluster (Figure 4B,E), the sulfonamide binding mode was further reinforced by a water-bridged hydrogen bond between the sulfonamide NH^−^ and the sidechain OH of T200, as well as by an H-bond network involving the sidechain NH_2_ of Q92. The dichlorobenzene ring was oriented toward a hydrophobic pocket defined by T69, L91, and the aliphatic chain of Q92, occasionally forming halogen bond with the backbone C=O of L91 (~2%).

In the second, more abundant cluster (Figure 4C,E), the C=S group of the hydrazine-1-carbothioamide linker engaged an H-bond with the backbone C=O of P201, either directly (~23% of the trajectory) or water-mediated (~17%). This interaction was further stabilized by an additional water molecule bridging the linker nitrogen to the sidechain OH of T200 (~11%). The dichlorobenzene ring was oriented within a more lipophilic pocket defined by L91, V121, V131, L141, and V143, establishing vdW contacts with L91 (~5%) and V131 (~25%), as well as halogen bonds with the backbone C=O of L91 (~2%), V121 (~1%), and H122 (~2%).

In CA XII, ligand **3l** quickly stabilized at an RMSD of ~5.7 Å and maintained this value throughout the 200 ns-long MD simulation (Figure 5A), giving rise to three main binding clusters (Figure 5B–D). The first cluster, which formed early, was also the most persistent (52.7%, Figure 5B,E), fluctuating around a second (13.7%, Figure 5C,E) and a third (22.6%, Figure 5D,E) cluster related conformations.

As in CA IX, the sulfonamide binding mode remained fully stable during the 200 ns MD trajectory (100%), both in terms of Zn^2+^ coordination and hydrogen bonding with T199 (Figure 5F). Additional vdW contacts between the benzenesulfonamide ring and H94 (~20%), V121 (~16%), and L198 (~69%) further stabilized its orientation within the active site.

In the first cluster (Figure 5B,F), the orientation of the hydrazine-1-carbothioamide linker was stabilized by a bidentate water-bridged hydrogen bond involving both the C=S and –N= groups with the backbone C=O of P201 (~46%). In some cases, the C=S group formed a direct H-bond with the backbone C=O of the residue (~5%). Moreover, water-mediated hydrogen-bonding networks connected the sulfonamide NH^−^ and the 2-chloro substituent to N62, Q92, and T200, as well as the 4-chloro substituent to S132 and N136.

In the second, less persistent cluster (Figure 5C,F), the benzenesulfonamide ring underwent distortion to engage in π–π stacking with H94 (~17%). This rearrangement enabled the 2-chloro atom to form a water-bridged hydrogen bond with the sidechain NH_2_ of Q92 and the protonated NH_3_^+^ group of the peculiar K67. Again, extended water-mediated hydrogen-bonding networks anchored the ligand to S65, T91, and P201.

In the third cluster (Figure 5D,F), the canonical orientation of the sulfonamide ring was restored, while the dichlorobenzene ring shifted toward residue K67, forming a strong π-cation interaction (~23%). The hydrazine-1-carbothioamide linker was further stabilized by a hydrogen-bonding network involving water molecules bridging the =N– group to the sidechains of T200 and Q92.

Overall, the selectivity of ligand **3l** can be rationalized by its ability to establish strong and stable interactions within the less sterically hindered active sites of CA IX and CA XII—sites that are more restricted in CA I/II due to the presence of A131/F131 and F91/I91. Additionally, unlike other ligands in the series, compound **3l** exploits both chlorine substituents to form weak H-bonds or halogen bonds with isoform-specific residues, contributing to its distinct binding profile.

### 2.4. Cytotoxicity Studies

The in vitro cytotoxicity of two representative carbothioamides **3a** the most selective inhibitor of hCA XII, and **3l**, the most potent inhibitor of both tumor-associated isoforms was evaluated in six human cancer cell lines (MDA-MB-231, MDA-MB-435, PANC-1, U-118, A2780 and A549) after 48 h of exposure, giving IC_50_ values in the 31.4–89.6 µM range, with **3l** generally more potent than **3a** in MDA-MB-231, U-118, A2780 and A549 cells (Table 3). These micromolar IC_50_ values are in line with those reported for many sulfonamide CA inhibitors with anticancer activity, which typically exhibit low- to mid-micromolar anti-proliferative.

Importantly, CA IX and CA XII are overexpressed in hypoxic, aggressive tumors where they regulate extracellular pH, promote invasion and metastasis, and correlate with poor prognosis. Selective inhibition of these tumor-associated isoforms is considered a promising anticancer strategy. In contrast, the ubiquitously expressed cytosolic isoforms CA I and CA II are regarded as off-target isoforms whose inhibition can sequester non-selective CAIs and contribute to systemic side effects, underscoring the need for isoform-selective agents.

Compounds **3a** and **3l** combine low-nanomolar inhibition of the tumor-associated CA isoforms (Table 2) with micromolar-range cytotoxicity (Table 3), placing their cellular effects within the range reported for other CA IX/XII-directed sulfonamides and indicating that the target isoforms can be efficiently inhibited at concentrations where non-specific cytotoxicity remains moderate.

It is worth to stress that synthesis of *N*-aryl-2-(4-sulfamoylphenyl)hydrazine-1-carbothioamides **3** is not reported in the literature. Moreover, our results demonstrate the potency on these compounds as effective and surprisingly selective inhibitors of CAs with potency for treatment of cancer.

## 3. Materials and Methods

### 3.1. Materials and Methods

All reagents and solvents were obtained from commercial suppliers (Sigma-Aldrich, Burlington, MA, USA, BLDpharm, Shanghai, China, TCI, Tokyo, Japan, Enamine, Kyiv, Ukraine, and Thermo Fisher Scientific, Waltham, MA, USA) and were used without any further purification. Analytical TLC was carried out on MERCK precoated silica gel F_254_ aluminum plates using a methanol–dichloromethane mixture as the eluent, and reaction progress was monitored under UV light. The ^1^H and ^13^C NMR spectra (see the Supporting Information) were recorded in DMSO-*d*_6_ on Bruker Avance spectrometer at 400 and 100 MHz, respectively, using deuterated dimethyl sulfoxide (DMSO-*d*_6_) and Me_4_Si (TMS) as internal standard at room temperature, and can be find in Supporting Information. High-resolution mass spectra (HRMS) were attained on a mass spectrometer with a Q-TOF micro mass analyzer using the electrospray ionization (ESI) technique.

MDA-MB-231, MDA-MB-435, PANC-1, U-118, A2780, and A549 human cancer cell lines were obtained from the American Type Culture Collection (ATCC, Manassas, VA, USA) and maintained according to the supplier’s recommendations. Dulbecco’s Modified Eagle Medium (DMEM) and fetal bovine serum (FBS) were purchased from ATCC (Manassas, VA, USA). Penicillin and streptomycin were obtained from Sigma-Aldrich (MA, USA). MTT reagent (3-(4,5-dimethylthiazol-2-yl)-2,5-diphenyltetrazolium bromide) was purchased from TCI EUROPE N.V. (Zwijndrecht, Belgium).

### 3.2. CA Inhibitory Assay

An Applied Photophysics stopped-flow instrument was used to assay the CA catalyzed CO_2_ hydration activity [31]. Phenol red (at a concentration of 0.2 mM) was used as an indicator, working at the absorbance maximum of 557 nm, with 20 mM Hepes (pH 7.4) as a buffer, and 20 mM Na_2_SO_4_ (to maintain constant ionic strength), following the initial rates of the CA-catalyzed CO_2_ hydration reaction for a period of 10–100 s. The CO_2_ concentrations ranged from 1.7 to 17 mM for the determination of the kinetic parameters and inhibition constants [6]. Enzyme concentrations ranged between 5–12 nM. For each inhibitor, at least six traces of the initial 5–10% of the reaction were used to determine the initial velocity. The uncatalyzed rates were determined in the same manner and subtracted from the total observed rates. Stock solutions of the inhibitor (0.1 mM) were prepared in distilled–deionized water and dilutions up to 0.01 nM were done thereafter with the assay buffer. Inhibitor and enzyme solutions were preincubated together for 15 min at room temperature prior to the assay, to allow for the formation of the E–I complex. The inhibition constants were obtained by non-linear least-squares methods using PRISM 3 and the Cheng–Prusoff equation as reported earlier and represent the mean from at least three different determinations. All CA isoforms were recombinant proteins obtained in house, as reported earlier [36,37].

### 3.3. In Silico Studies

The crystal structures of hCA IX (PDB 5FL4) [34] and hCA XII (PDB 1JD0) [35] were downloaded from the Protein Data Bank (RCSB.org) [38] and prepared using the Protein Preparation module implemented in the Maestro Schrödinger suite [39], assigning bond orders, adding hydrogens, deleting water molecules, and optimizing H-bonding networks. Finally, energy minimization with a Root Means Square Deviation (RMSD) value of 0.30 was applied using an Optimized Potential for Liquid Simulation (OPLS4) force field [39,40,41,42]. The 3D ligand structures were prepared by Maestro [39] (a) and evaluated for their ionization states at pH 7.3 ± 1.0 with Epik [39] (b). The conjugate gradient method in Macromodel [39] (c) was used for energy minimization. Grids for docking were centered on the centroid of the complexed ligand. 3D ligand structures were prepared by Maestro [39] (a). Docking studies were carried out with the program Glide [39] (d) using the standard precision (SP) mode. Molecular dynamics (MD) simulations were performed using Desmond Molecular Dynamics System (v.7.0) [39] (e) and OPLS4 force field. The MD simulation protocol included a starting relaxation step followed by a final production phase of 200 ns. Protein RMSD, ligand RMSD/RMSF (Root Mean Square Fluctuation), ligand torsions evolution, and occurrence of intermolecular H-bonds and hydrophobic contacts were provided by the Simulation Interaction Diagram (SID) implemented in Maestro, along with the production phase of the MD simulation. The 1000 frames resulting from MDs were clustered using the Conformer Cluster tool implemented in the Schrödinger suite into 10 clusters. Figures were generated with Maestro and Chimera [39,43].

### 3.4. Cell Culture and MTT Cell Viability Assay

Cells were seeded at a density of 1000 cells per well in 96-well plates and cultured in DMEM (MDA-MB-231, MDA-MB-435, PANC-1, U-118, A549) or RPMI medium (A2780) supplemented with 10% (*v*/*v*) FBS, 100 µg/mL penicillin, and 100 µg/mL streptomycin. Cultures were maintained at 37 °C in a humidified atmosphere containing 5% CO_2_. After 24 h of incubation to allow cell attachment, the medium was replaced with fresh medium containing the tested compounds at final concentrations ranging 3.9–500 µM (*n* = 6 wells per concentration). Following 48 h of exposure, the medium was aspirated and cell viability was assessed using the MTT assay. Cells incubated with complete growth medium in the absence of test compound served as the negative control, cells treated with 0.1% saponin for 30 min prior to the MTT assay served as the positive (cytotoxic) control.

MTT results were analyzed using GraphPad Prism v.10.3.1 (GraphPad Software, San Diego, CA, USA) and IC_50_ values were obtained by fitting the viability data with a four-parameter logistic (4PL) sigmoidal non-linear regression model.

## 4. Conclusions

CAs are well known to be involved in various diseases such as cancer, glaucoma, brain edema, altitude sickness, epilepsy, and obesity have become specific targets in drug designing. The potential of (thio)urea-substituted benzene sulfonamide derivatives as isoform selective CAIs led us to synthesize a novel series of twelve *N*-aryl-2-(4-sulfamoylphenyl)hydrazine-1-carbothioamides **3** along with two new *N*-aryl-2-(4-sulfamoylphenyl)hydrazine-1-carboxamide derivatives **5** to evaluate their effect on four hCA isozymes (hCA I, II, IX and XII). Kinetic studies revealed that all compounds exhibited strong inhibition against the tested isozymes in the nanomolar range. Compound **3i** demonstrated the highest inhibition of hCA I activity but showed poor selectivity towards the other three isozymes. Compound **3h** displayed superior selectivity against hCA II vs. hCA I (hCA I/II = 37), while it showed 2.5-fold higher inhibitory activity against this isozyme compared to AAZ. Among the tested compounds, compound **3l** (with *K_i_* = 32.1) displayed much better selectivity against hCA IX vs. hCA I, II, and XII compared to the standard drug. On the other hand, compound **3a** displayed the most potent inhibition against hCA XII with a *K_i_* of 6.8 nM, comparable to that of AAZ, while demonstrating significantly greater selectivity over off-target isoforms and over the other tumor-associated isozyme (hCAIX/XII = 20) compared to AAZ (hCA IX/XII = 4.5). The combined docking and MD studies revealed that the high selectivity of ligand **3l** toward tumor-associated isoforms arises from its ability to establish strong and stable interactions within the less sterically hindered active sites of CA IX and CA XII. In addition, both of its distinctive chlorine substituents contribute by forming weak hydrogen or halogen bonds with isoform-specific residues. Based on these findings, these compounds could serve as a starting point for the development of isoform-selective hCAIs, particularly for highly effective and selective molecules targeting cytosolic hCA II and tumor-associated hCA XII.

## Figures and Tables

**Table 1:** CA inhibition data with *N*-aryl-2-(4-sulfamoylphenyl)hydrazine-1-carbothioamides (**3a**–**3l**) and acetazolamide (AAZ) as a standard drug, by the stopped-flow CO_2_ hydrase assay.

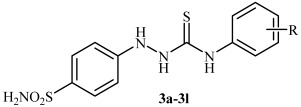						
Compound	R	*K*_i_ (nM) ^[a]^				Selectivity Index ^[b]^
		hCA I	hCA II	hCA IX	hCA XII	hCA XII/hCAIX
**3a**	H	533.9	56.6	133.3	6.8	19.6
**3b**	4-Me	95.6	6.1	251.0	19.7	12.7
**3c**	4-^i^Pr	74.5	6.6	198.3	9.0	22.0
**3d**	4-Br	187.7	7.6	81.9	6.6	12.4
**3e**	4-Cl	92.9	7.7	27.5	23.1	1.2
**3f**	4-NO_2_	27.6	7.1	122.0	5.5	22.2
**3g**	3-Me	77.7	66.5	73.2	9.3	7.9
**3h**	3-Br	176.6	4.8	31.2	17.6	1.8
**3i**	3-Cl	7.7	4.3	23.9	7.9	3.0
**3j**	3-CN	87.6	5.7	122.5	7.2	17.0
**3k**	2-Br	354.9	43.7	68.7	6.3	10.9
**3l**	2,4-(Cl)_2_	699.4	78.8	32.1	9.5	3.4
**AAZ**	-	250.0	12.1	25.7	5.7	4.5

^[a]^ Mean from 3 different assays, by a stopped flow technique (errors were in the range of ±5–10% of the reported values). ^[b]^ Calculated as the ratio *K*_i_ (CA XII)/*K*_i_ (CA IX).

**Table 2:** CA inhibition data with *N*-aryl-2-(4-sulfamoylphenyl)hydrazine-1-carboxamides (**5a**,**b**) and acetazolamide (AAZ) as a standard drug, by the stopped-flow CO_2_ hydrase assay.

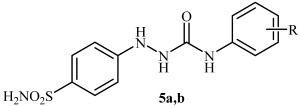						
Compound	R	*K*_i_ (nM) ^[a]^				Selectivity Index ^[b]^
		hCA I	hCA II	hCA IX	hCA XII	hCA XII/hCA IX
**5a**	H	749.9	92.8	255.0	8.2	31.1
**5b**	4-Me	86.3	9.1	278.8	6.6	42.2
**AAZ**	-	250.0	12.1	25.7	5.7	4.5

^[a]^ Mean from 3 different assays, by a stopped flow technique (errors were in the range of ±5–10% of the reported values). ^[b]^ Calculated as the ratio *K*_i_ (CA XII)/*K*_i_ (CA IX).

**Table 3:** In vitro cytotoxicity (IC_50_, µM) of *N*-aryl-2-(4-sulfamoylphenyl)hydrazine-1-carbothioamides **3a** and **3l** after 48 h of incubation.

Compound	Cell Line					
	MDA-MB-231	MDA-MB-435	PANC-1	U-118	A2780	A549
**3a**	50.8 ± 1.5	80.1 ± 7.0	87.1 ± 3.3	85.3 ± 6.0	53.9 ± 4.6	81.0 ± 7.0
**3l**	31.4 ± 3.0	89.6 ± 11.4	83.0 ± 11.2	47.2 ± 3.1	41.1 ± 7.5	38.4 ± 2.3

## Data Availability

The original contributions presented in this study are included in the article/Supplementary Materials. Further inquiries can be directed to the corresponding author.

## Supplementary Materials

The following supporting information can be downloaded at: https://www.mdpi.com/article/10.3390/ph19010151/s1, Experimental procedures for the synthesis of inhibitors; ^1^H NMR and ^13^C NMR spectra of inhibitors; High resolution mass spectra of inhibitors.
