# Supplementary material for: Investigation on N-Aryl-2-(4-sulfamoylphenyl)hydrazine-1-carbothioamide as Human Carbonic Anhydrases Inhibitors
Source: Pharmaceuticals (Basel). 2026 Jan 14;19(1):151. doi: 10.3390/ph19010151 (PMC12844790; doi:10.3390/ph19010151)
Supplement: Supplementary file 1 [file pharmaceuticals-19-00151-s001.zip › pharmaceuticals-4063105-supplementary.pdf]

Investigation on *N*-Aryl-2-(4-sulfamoylphenyl)hydrazine-1-carbothioamide as Human Carbonic Anhydrases Inhibitors

Morteza Abdoli [1], Andrea Angeli [2], Alessandro Bonardi [2,3], Paola Gratteri [3], Ludmila Jackevica [1], Antons Sizovs [1,4], Claudiu T. Supuran [2] and Raivis Žalubovskis [1,5,*]

[1] Latvian Institute of Organic Synthesis, LV-1006 Riga, Latvia; morteza.abdoli@osi.lv (M.A.); ludmila@farm.osi.lv (L.J.); antons.sizovs@farm.osi.lv (A.S.)
[2] Department of Neurofarba, Università degli Studi di Firenze, 50019 Florence, Italy; andrea.angeli@unifi.it (A.A.); alessandro.bonardi@unifi.it (A.B.); claudiu.supuran@unifi.it (C.T.S.)
[3] Pharmaceutical and Nutraceutical Section and Laboratory of Molecular Modeling Cheminformatics & QSAR, Department of NEUROFARBA, University of Florence, Via U. Schiff 6, Sesto Fiorentino, 50019 Florence, Italy; paola.gratteri@unifi.it
[4] Baltic Biomaterials Centre of Excellence, Headquarters at Riga Technical University, LV-1048 Riga, Latvia
[5] Institute of Chemistry and Chemical Technology, Faculty of Natural Sciences and Technology, Riga Technical University, LV-1048 Riga, Latvia
* Correspondence: raivis@osi.lv

Supporting information

1. Synthesis

1.1. General procedure (A) for the synthesis of N-aryl-2-(4-sulfamoylphenyl)hydrazine-1-carbothioamides (**3a**-**3l**)

The appropriate aryl isothiocyanate (1.0 equiv., 1.788 mmol) and triethylamine (1.0 equiv., 1.788 mmol, 0.249 mL) were added to a suspension of (4-sulfamoylphenyl)hydrazine hydrochloride (1.0 equiv., 1.788 mmol, 400 mg) in MeCN (10 mL) at 0 °C. The mixture was stirred at the same temperature for 3 h. Then brine was added to the reaction mixture, and it was extracted with EtOAc (3 × 30 mL). The collected organic phases were dried over anhydrous Na_2SO_4, filtered, and concentrated under reduced pressure. The residue was dissolved in ^{i}PrOH (10 mL) and triturated with hexanes (100 mL). The formed precipitate was collected and further purified by silica gel column chromatography (MeOH:DCM, 1:10).

1.2. General procedure (B) for the synthesis of *N*-aryl-2-(4-sulfamoylphenyl)hydrazine-1-carboxamides **5a** and **5b**

The appropriate aryl isocyanate (1.0 equiv., 1.788 mmol) and triethylamine (1.0 equiv., 1.788 mmol, 0.249 mL) were added to a suspension of (4-sulfamoylphenyl)hydrazine hydrochloride (1.0 equiv., 1.788 mmol, 400 mg) in MeCN (10 mL) at 0 °C. The mixture was stirred at above temperature for 3 h and then allowed to warm to 20 °C and stirred for additional 12 h. Then, the reaction was stopped and the solvent was evaporated under reduced pressure and the resulting solid was recrystallized from ethanol to get pure product.

N-Phenyl-2-(4-sulfamoylphenyl)hydrazine-1-carbothioamide (**3a**)

Following the general procedure (A), compound **3a** was obtained as a white solid. (300 mg, 52% yield). **R$_f$**: 0.35 (MeOH/DCM = 1:10).

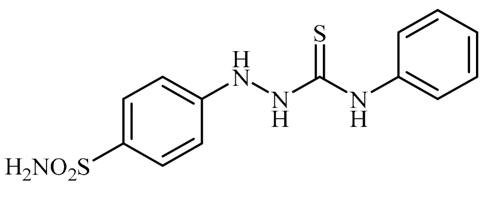

^{1}H NMR (400 MHz, DMSO-*d$_6$*) δ = 6.80–6.84 (m, 2H), 7.09 (s, 2H), 7.12–7.15 (m, 1H), 7.28–7.32 (m, 2H), 7.47-7.49 (m, 2H), 7.66-7.68 (m, 2H), 8.59 (s, 1H), 9.82 (s, 1H), 9.87 (s, 1H) ppm. **^{13}C NMR** (100 MHz, DMSO-*d$_6$*) δ = 112.0, 125.0, 125.6, 127.2, 128.0, 134.6, 139.2, 150.9, 181.4 ppm. **HRMS** (ESI) $[M + H]^+$: *m*/*z* calcd for ($C_{13}H_{15}N_4O_2S_2$) 323.0636. Found 323.0646.

2-(4-Sulfamoylphenyl)-*N*-(*p*-tolyl)hydrazine-1-carbothioamide (**3b**)

Following the general procedure (A), compound **3b** was obtained as a white solid. (219 mg, 36% yield). **R$_f$**: 0.34 (MeOH/DCM = 1:10).

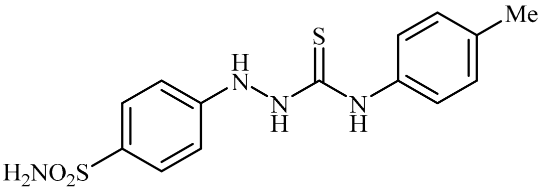

^{1}H NMR (400 MHz, DMSO-*d6*) δ = 2.27 (s, 3H), 6.80–6.83 (m, 2H), 7.08–7.11 (m, 4H), 7.32–7.34 (m, 2H), 7.65-7.67 (m, 2H), 8.57 (s, 1H), 9.75 (s, 1H), 9.78 (s, 1H) ppm. **^{13}C NMR** (100 MHz, DMSO-*d6*) δ = 20.5, 111.9, 125.5, 127.1, 128.4, 134.1, 134.4, 150.9, 181.4 ppm. **HRMS** (ESI) $[M + H]^+$: *m/z* calcd for ($C_{14}H_{17}N_4O_2S_2$) 337.0793. Found 337.0801.

N-(4-isopropylphenyl)-2-(4-sulfamoylphenyl)hydrazine-1-carbothioamide (**3c**)

Following the general procedure (A), compound **3c** was obtained as a white solid. (112 mg, 17% yield). **R$_f$**: 0.42 (MeOH/DCM = 1:10).

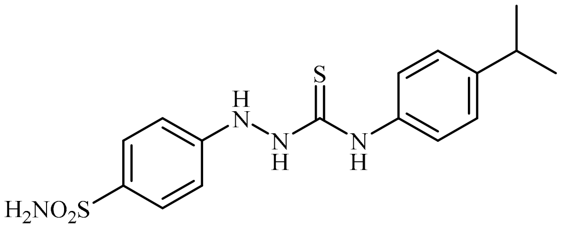

^{1}H NMR (400 MHz, DMSO-*d6*) δ = 1.18 (d, *J*= 7.0 Hz, 6 H), 2.80-2.91 (m, 1H), 6.79–6.83 (m, 2H), 7.09 (s, 2H), 7.15–7.17 (m, 2H), 7.35-7.38 (m, 2H), 7.65-7.67 (m, 2H), 8.57 (s, 1H), 9.76 (s, 1H), 9.78 (s, 1H) ppm. **^{13}C NMR** (100 MHz, DMSO-*d6*) δ = 24.4, 33.4, 112.4, 125.9, 126.2, 127.6, 134.9, 137.2, 145.6, 151.4, 181.8 ppm. **HRMS** (ESI) $[M + H]^+$: *m/z* calcd for ($C_{16}H_{21}N_4O_2S_2$) 365.1106. Found 365.1116.

N-(4-Bromophenyl)-2-(4-sulfamoylphenyl)hydrazine-1-carbothioamide (**3d**)

Following the general procedure (A), compound **3d** was obtained as a light pink solid. (380 mg, 53% yield). **R$_f$**: 0.35 (MeOH/DCM = 1:10).

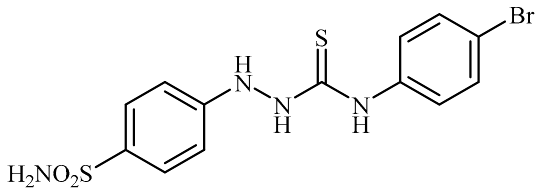

^{1}H NMR (400 MHz, DMSO-*d6*) δ = 6.80–6.82 (m, 2H), 7.09 (s, 2H), 7.44–7.49 (m, 4H), 7.66–7.68 (m, 2H), 8.61 (s, 1H), 9.92 (s, 1H), 9.96 (s, 1H) ppm. **^{13}C NMR** (100 MHz, DMSO-*d6*) δ = 112.0, 126.6, 127.1, 127.5, 130.7, 130.9, 138.6, 150.8, 181.2 ppm. **HRMS** (ESI) $[M + H]^+$: *m/z* calcd for ($C_{13}H_{14}N_4O_2S_2Br$) 400.9742. Found 400.9748.

N-(4-Chlorophenyl)-2-(4-sulfamoylphenyl)hydrazine-1-carbothioamide (**3e**)

Following the general procedure (A), compound **3e** was obtained as a white solid. (285 mg, 45% yield). **R$_f$**: 0.36 (MeOH/DCM = 1:10).

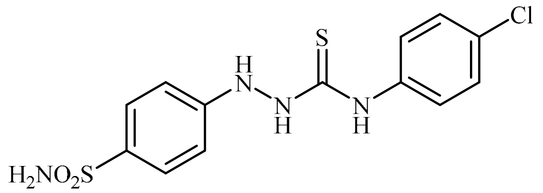

^{1}H NMR (400 MHz, DMSO-*d$_6$*) δ = 6.79–6.83 (m, 2H), 7.09 (s, 2H), 7.34–7.37 (m, 2H), 7.51–7.55 (m, 2H), 7.65–7.69 (m, 2H), 8.61 (s, 1H), 9.91 (s, 1H), 9.97 (s, 1H) ppm. **^{13}C NMR** (100 MHz, DMSO-*d$_6$*) δ = 112.0, 127.1, 127.8, 128.9, 130.7, 134.6, 138.2, 150.8, 181.4 ppm. **HRMS** (ESI) [M + H]$^+$: *m/z* calcd for ($C_{13}H_{14}N_4O_2S_2Cl$) 357.0247. Found 357.0250.

N-(4-nitrophenyl)-2-(4-sulfamoylphenyl)hydrazine-1-carbothioamide (**3f**)

Following the general procedure (A), compound **3f** was obtained as a white solid. (167 mg, 25% yield). **R$_f$**: 0.37 (MeOH/DCM = 1:10).

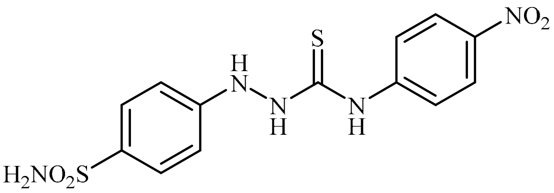

^{1}H NMR (400 MHz, DMSO-*d$_6$*) δ = 6.82–6.85 (m, 2H), 7.10 (s, 2H), 7.67–7.69 (m, 2H), 7.97–7.99 (m, 2H), 8.16–8.20 (m, 2H), 8.70 (s, 1H), 10.23 (s, 1H), 10.34 (s, 1H) ppm. **^{13}C NMR** (100 MHz, DMSO-*d$_6$*) δ = 112.2, 123.6, 124.4, 127.2, 135.0, 143.3, 145.7, 150.6, 181.0 ppm. **HRMS** (ESI) [M + H]$^+$: *m/z* calcd for ($C_{13}H_{14}N5O_4S_2$) 368.0487. Found 368.0494.

2-(4-sulfamoylphenyl)-*N*-(*m*-tolyl)hydrazine-1-carbothioamide (**3g**)

Following the general procedure (A), compound **3g** was obtained as a pink solid. (156 mg, 26% yield). **R$_f$**: 0.41 (MeOH/DCM = 1:10).

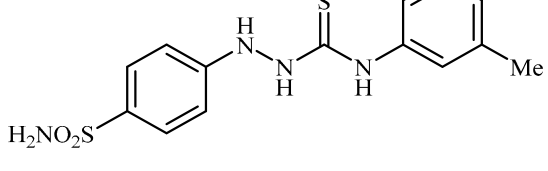

^{1}H NMR (400 MHz, DMSO-*d$_6$*) δ = 2.27 (s, 3H), 6.79–6.83 (m, 2H), 6.94–6.96 (m, 1H), 7.09 (s, 2H), 7.16–7.20 (m, 1H), 7.27–7.32 (m, 2H), 7.65–7.68 (m, 2H), 8.58 (s, 1H), 9.79 (s, 2H) ppm. **^{13}C NMR** (100 MHz, DMSO-*d$_6$*) δ = 21.0, 112.0, 122.6, 125.7, 126.0, 127.2, 127.8, 134.5, 137.2, 139.0, 150.9, 181.3 ppm. **HRMS** (ESI) [M + H]$^+$: *m/z* calcd for ($C_{14}H_{17}N_4O_2S_2$) 337.0793. Found 337.0794.

N-(3-Bromophenyl)-2-(4-sulfamoylphenyl)hydrazine-1-carbothioamide (**3h**)

Following the general procedure (A), compound **3h** was obtained as a white solid. (257 mg, 36% yield). **R$_f$**: 0.41 (MeOH/DCM = 1:10).

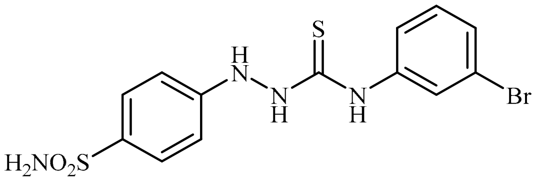

^{1}H NMR (400 MHz, DMSO-*d$_6$*) δ = 6.80–6.83 (m, 2H), 7.09 (s, 2H), 7.24–7.34 (m, 2H), 7.53–7.56 (m, 1H), 7.66–7.68 (m, 2H), 7.83–7.84 (m, 1H), 8.62 (s, 1H), 9.97 (s, 1H), 10.01 (s, 1H) ppm. **^{13}C NMR** (100 MHz, DMSO-*d$_6$*) δ = 112.5, 120.8, 124.6, 127.6, 127.9, 128.1, 130.2, 135.1, 141.3, 151.2, 181.7 ppm. **HRMS** (ESI) $[M + H]^+$: *m*/*z* calcd for ($C_{13}H_{14}N_4O_2S_2Br$) 400.9742. Found 400.9746.

N-(3-chlorophenyl)-2-(4-sulfamoylphenyl)hydrazine-1-carbothioamide (**3i**)

Following the general procedure (A), compound **3i** was obtained as a white solid. (225 mg, 35% yield). **R$_f$**: 0.36 (MeOH/DCM = 1:10).

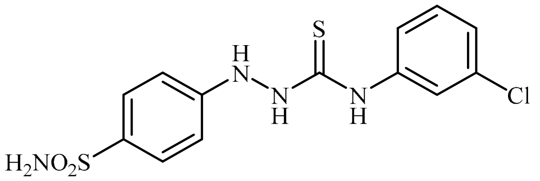

^{1}H NMR (400 MHz, DMSO-*d$_6$*) δ = 6.80–6.83 (m, 2H), 7.10 (s, 2H), 7.17–7.20 (m, 1H), 7.30–7.34 (m, 1H), 7.48–7.51 (m, 1H), 7.66–7.68 (m, 2H), 7.71–7.72 (m, 1H), 8.62 (s, 1H), 9.97 (s, 1H), 10.01 (s, 1H) ppm. **^{13}C NMR** (100 MHz, DMSO-*d$_6$*) δ = 112.1, 123.8, 124.6, 124.9, 127.2, 129.5, 132.0, 134.7, 140.7, 150.8, 181.3 ppm. **HRMS** (ESI) $[M + H]^+$: *m*/*z* calcd for ($C_{13}H_{14}N_4O_2S_2Cl$) 357.0247. Found 357.0245.

N-(3-Cyanophenyl)-2-(4-sulfamoylphenyl)hydrazine-1-carbothioamide (**3j**)

Following the general procedure (A), compound **3j** was obtained as a white solid. (189 mg, 30% yield). **R$_f$**: 0.33 (MeOH/DCM = 1:10).

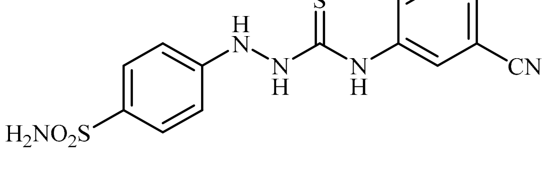

^{1}H NMR (400 MHz, DMSO-*d$_6$*) δ = 6.81–6.85 (m, 2H), 7.10 (s, 2H), 7.49–7.52 (m, 1H), 7.58–7.60 (m, 1H), 7.67–7.69 (m, 2H), 7.85–7.88 (m, 1H), 8.04 (s, 1H), 10.07 (s, 1H), 10.15 (s, 1H) ppm. **^{13}C NMR** (100 MHz,

DMSO-d_6) δ = 110.7, 112.2, 118.7, 127.2, 128.5, 128.6, 129.4, 130.3, 134.9, 140.2, 150.7, 181.4 ppm. **HRMS** (ESI) $[M + H]^+$: *m*/*z* calcd for ($C_{14}H_{14}N_5O_2S_2$) 348.0589. Found 348.0588.

N-(2-Bromophenyl)-2-(4-sulfamoylphenyl)hydrazine-1-carbothioamide (**3k**)

Following the general procedure (A), compound **3k** was obtained as a white solid. (215 mg, 30% yield). **R$_f$**: 0.44 (MeOH/DCM = 1:10).

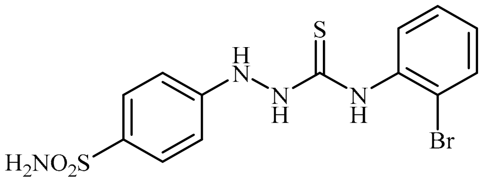

^{1}H NMR (400 MHz, DMSO-d_6) δ = 6.88–6.90 (m, 2H), 7.10 (s, 2H), 7.16–7.20 (m, 1H), 7.35–7.39 (m, 1H), 7.55–7.58 (m, 1H), 7.62–7.64 (m, 1H), 7.67–7.69 (m, 2H), 8.70 (s, 1H), 9.75 (s, 1H), 9.99 (s, 1H) ppm. **^{13}C NMR** (100 MHz, DMSO-d_6) δ = 112.3, 121.7, 127.1, 127.7, 128.0, 130.1, 132.4, 134.8, 138.0, 150.8, 181.9 ppm. **HRMS** (ESI) $[M + H]^+$: *m*/*z* calcd for ($C_{13}H_{14}N_4O_2S_2Br$) 400.9742. Found 400.9747.

N-(2,4-Dichlorophenyl)-2-(4-sulfamoylphenyl)hydrazine-1-carbothioamide (**3l**)

Following the general procedure (A), compound **3l** was obtained as a white solid. (254 mg, 36% yield). **R$_f$**: 0.39 (MeOH/DCM = 1:10).

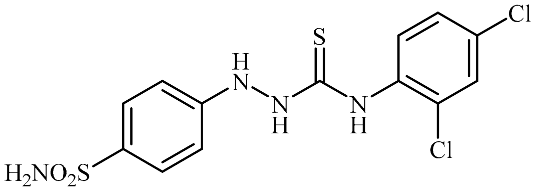

^{1}H NMR (400 MHz, DMSO-d_6) δ = 6.85–6.87 (m, 2H), 7.11 (s, 2H), 7.39–7.42 (m, 1H), 7.50–7.52 (m, 1H), 7.63–7.69 (m, 3H), 8.71 (s, 1H), 9.78 (s, 1H), 10.07 (s, 1H) ppm. **^{13}C NMR** (100 MHz, DMSO-d_6) δ = 112.2, 127.2, 127.3, 128.8, 131.3, 131.7, 132.3, 134.9, 136.0, 150.8, 182.2 ppm. **HRMS** (ESI) $[M + H]^+$: *m*/*z* calcd for ($C_{13}H_{13}N_4O_2S_2Cl_2$) 390.9857. Found 390.9861.

N-Phenyl-2-(4-sulfamoylphenyl)hydrazine-1-carboxamide (**5a**)

Following the general procedure (B), compound **5a** was obtained as a white solid. (309 mg, 56% yield). **R$_f$**: 0.28 (MeOH/DCM = 1:10).

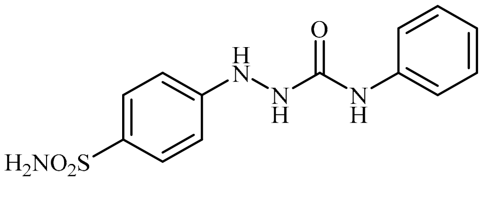

^{1}H NMR (400 MHz, DMSO-*d6*) δ = 6.83 (d, *J*= 8.6 Hz, 2H), 6.92–6.96 (m, 1H), 7.03 (s, 2H), 7.21–7.25 (m, 2H), 7.49–7.51 (m, 2H), 7.62 (d, *J*= 8.6 Hz, 2H), 8.29 (s, 2H), 8.75 (s, 1H) ppm. **^{13}C NMR** (100 MHz, DMSO-*d6*) δ = 111.3, 118.8, 121.9, 127.1, 128.5, 133.5, 139.7, 152.4, 156.1 ppm. **HRMS** (ESI) $[M + H]^{+}$: *m*/*z* calcd for ($C_{13}H_{15}N_4O_3S$) 307.0865. Found 307.0868.

2-(4-Sulfamoylphenyl)-*N*-(*p*-tolyl)hydrazine-1-carboxamide (**5b**)

Following the general procedure (B), compound **5b** was obtained as a white solid. (177 mg, 31% yield). **R_f**: 0.29 (MeOH/DCM = 1:10).

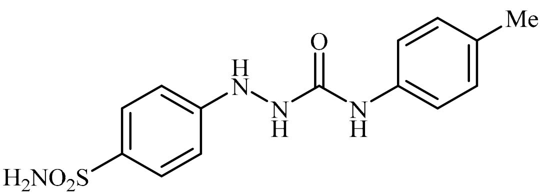

^{1}H NMR (400 MHz, DMSO-*d6*) δ = 2.22 (s, 3H), 6.81–6.83 (m, 2H), 7.02–7.04 (m, 4H), 7.37–7.39 (m, 2H), 7.61–7.63 (m, 2H), 8.24 (m, 1H), 8.27 (s, 1H), 8.65 (s, 1H) ppm. **^{13}C NMR** (100 MHz, DMSO-*d6*) δ = 20.3, 111.3, 118.9, 127.1, 128.9, 130.7, 135.5, 137.1, 152.4, 156.2 ppm. **HRMS** (ESI) $[M + H]^{+}$: *m*/*z* calcd for ($C_{14}H_{17}N_4O_3S$) 321.1021. Found 321.1028.

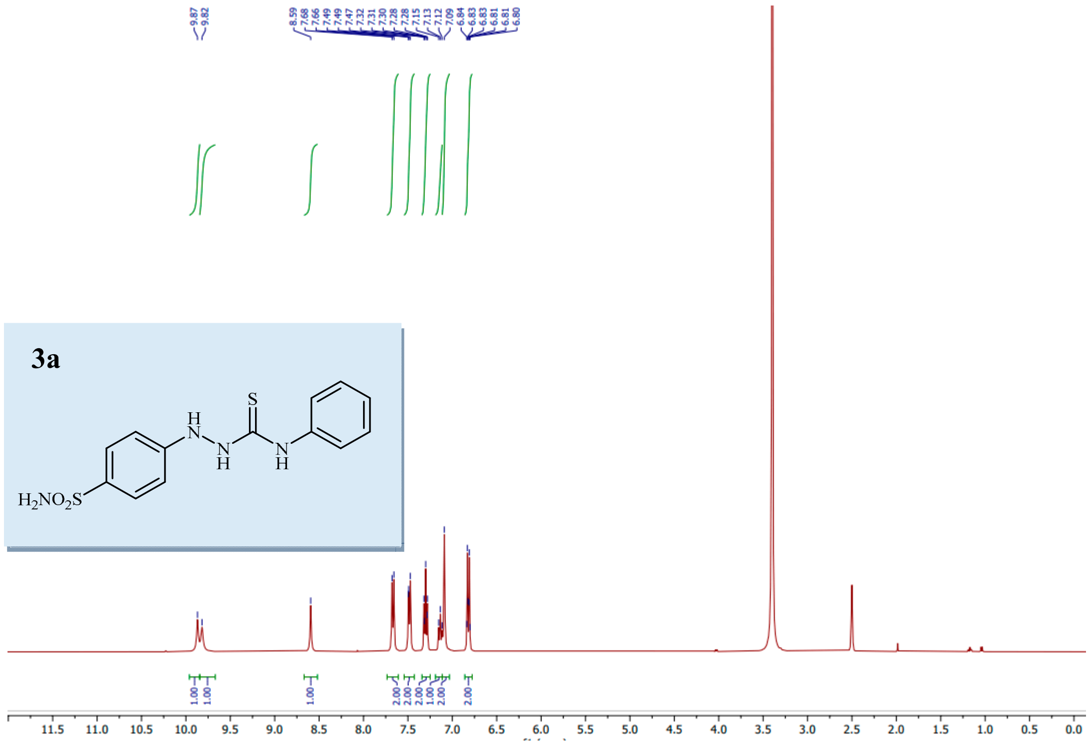

^{1}H NMR spectrum of compound **3a**.

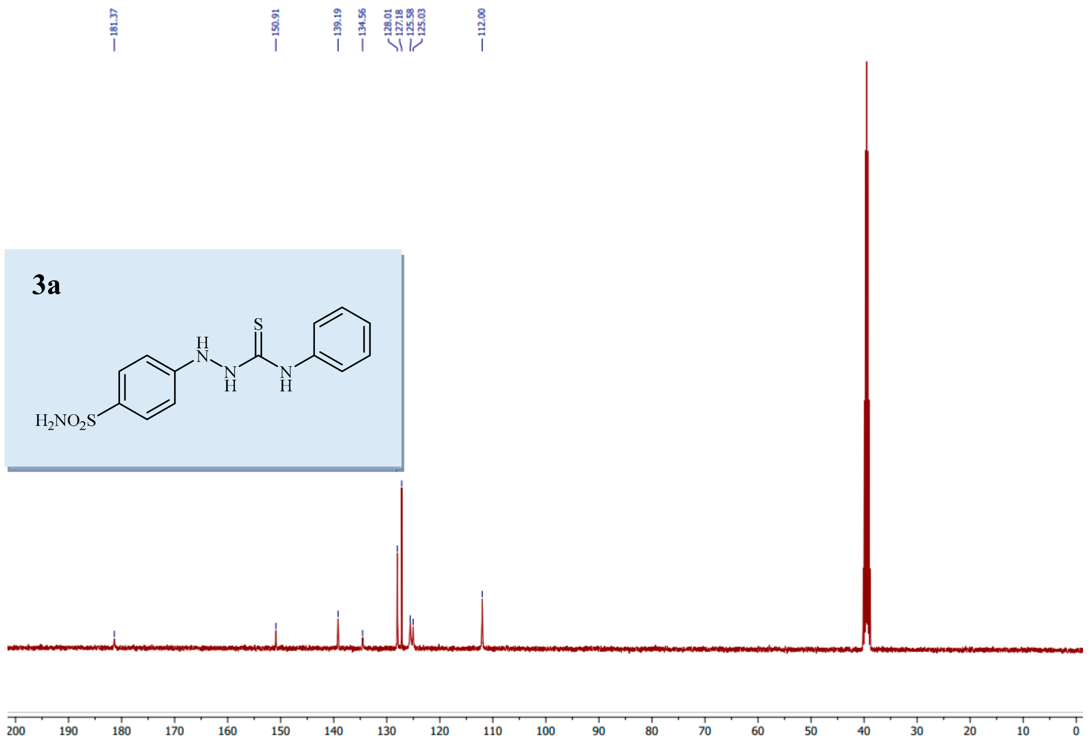

^{13}C NMR spectrum of compound **3a**.

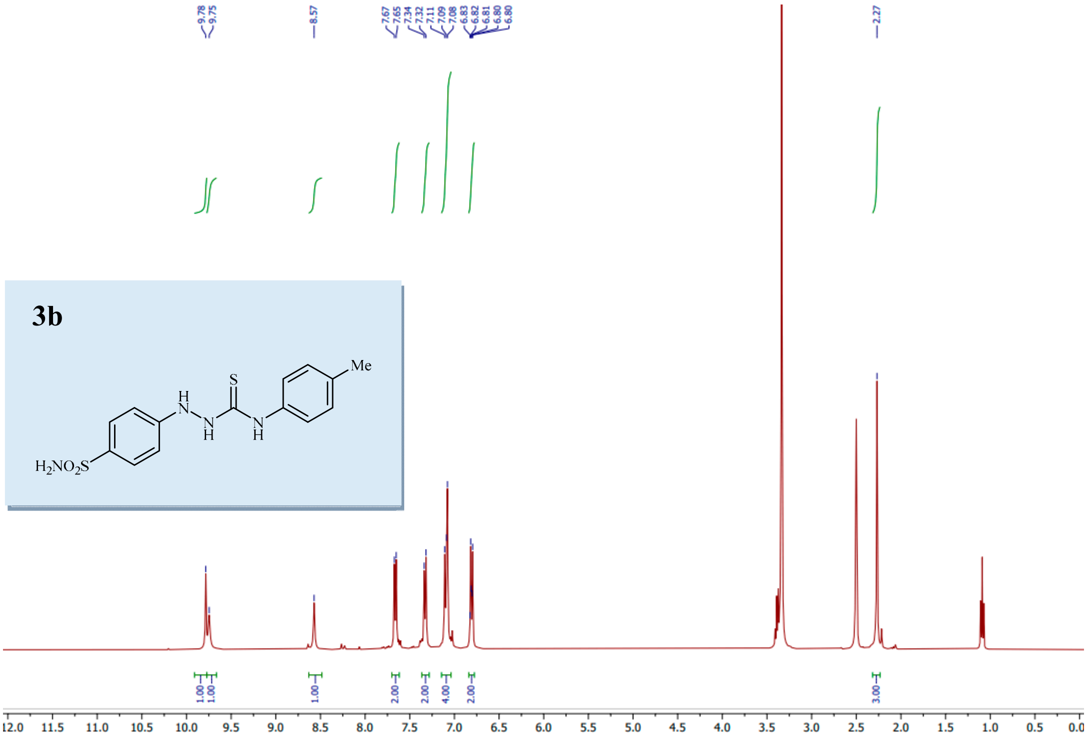

^{1}H NMR spectrum of compound **3b**.

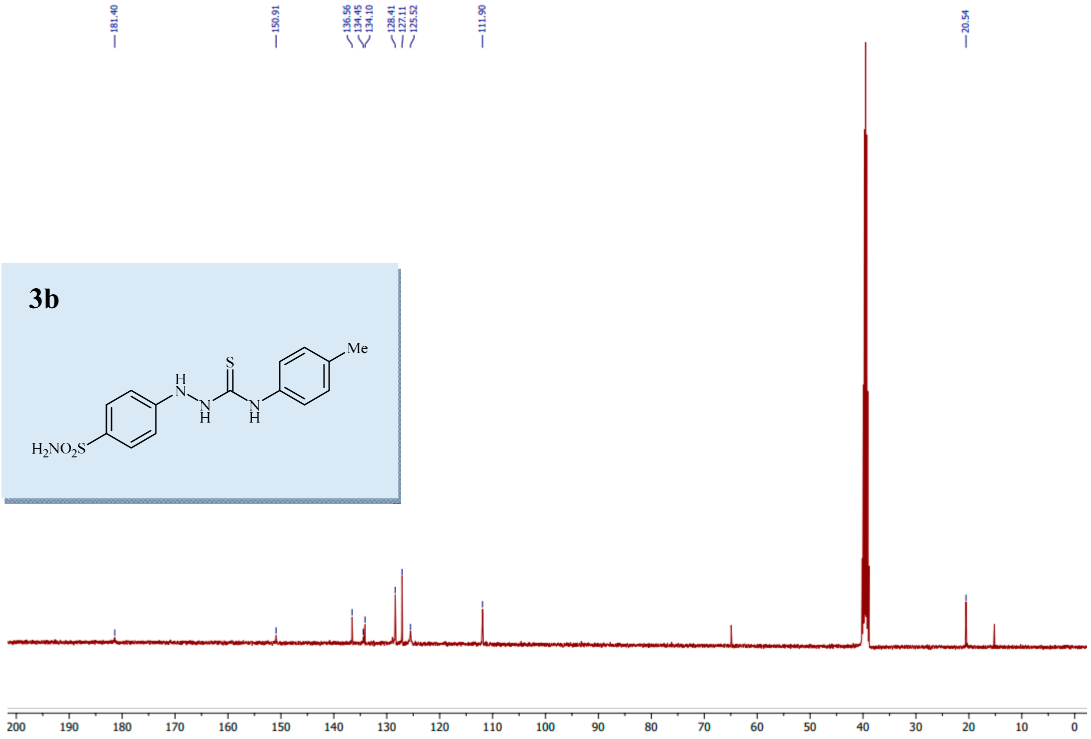

^{13}C NMR spectrum of compound **3b**.

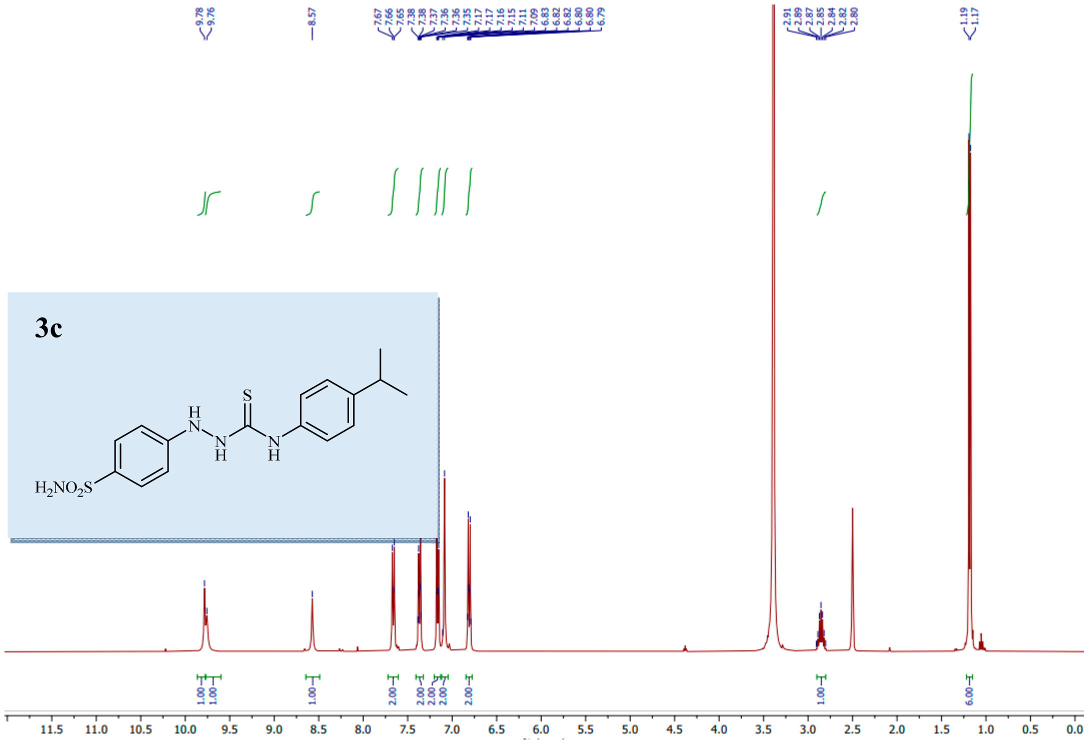

^{1}H NMR spectrum of compound **3c**.

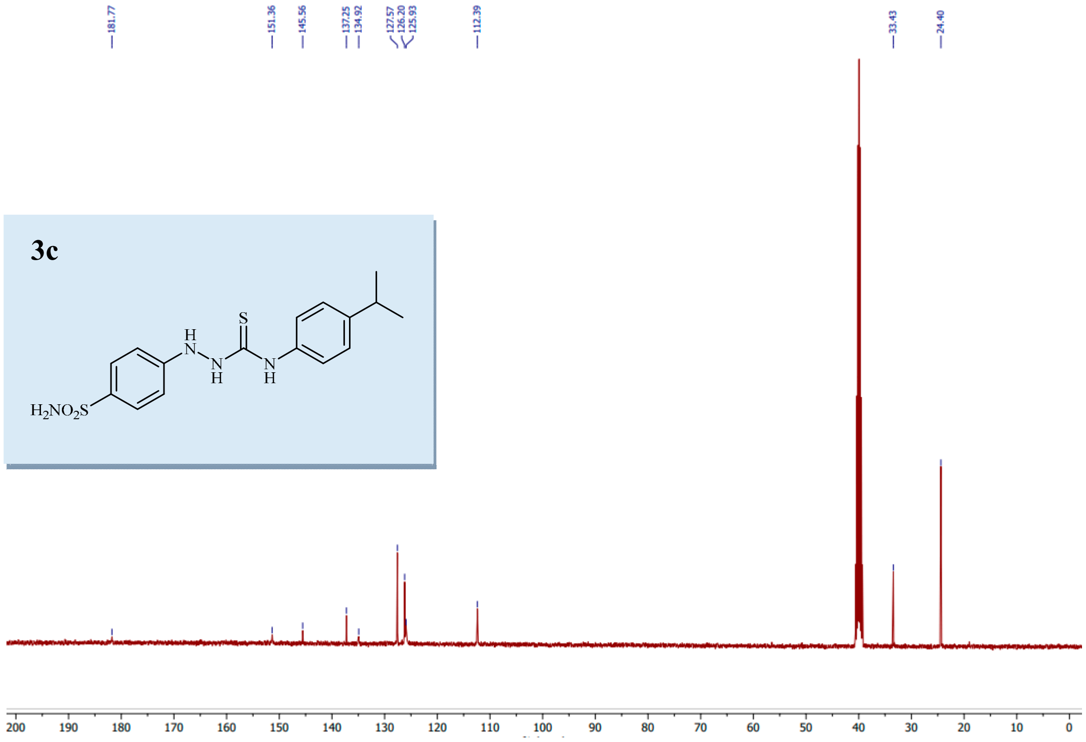

^{13}C NMR spectrum of compound **3c**.

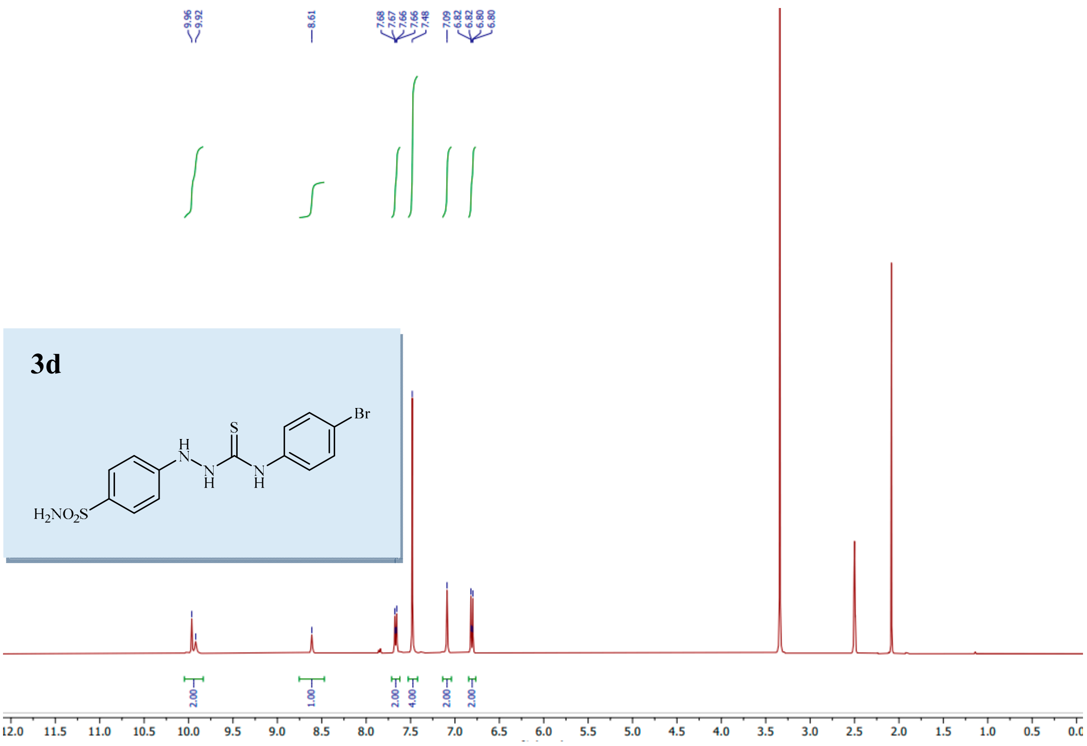

^{1}H NMR spectrum of compound **3d**.

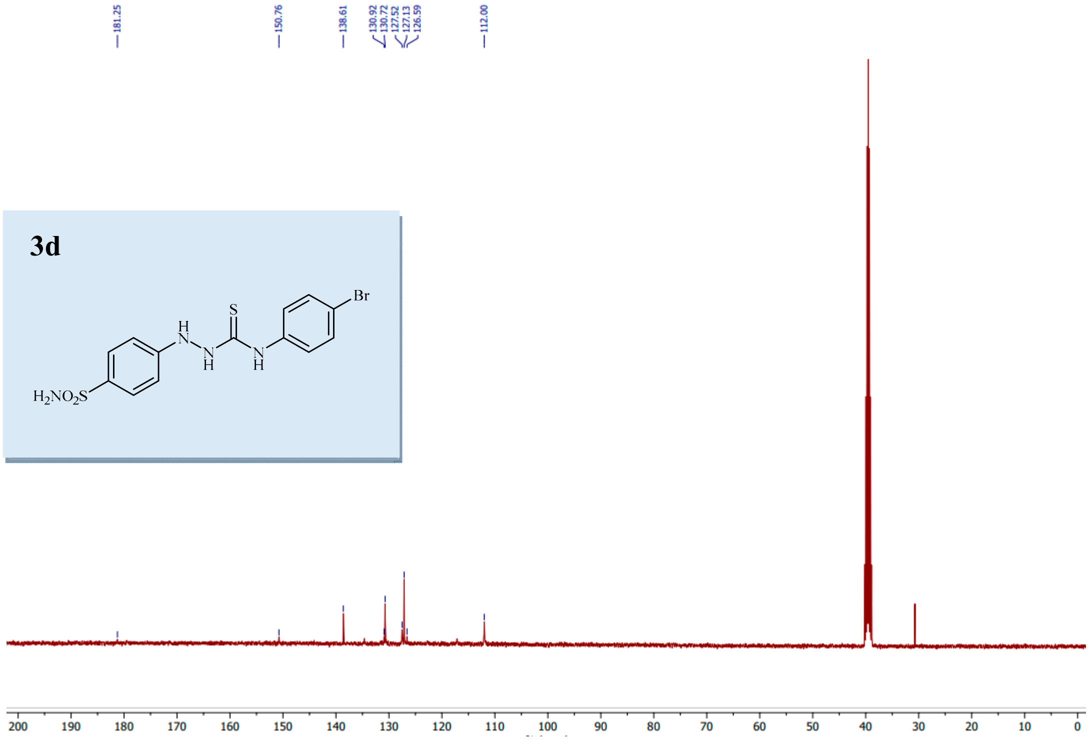

^{13}C NMR spectrum of compound **3d**.

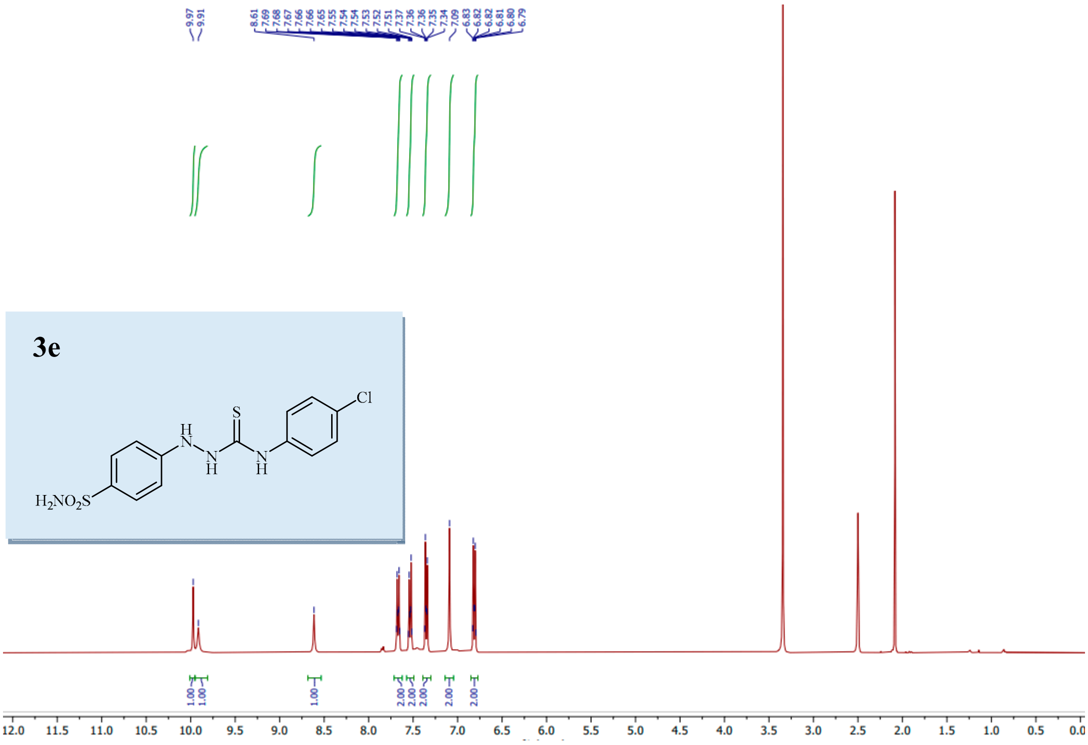

^{1}H NMR spectrum of compound **3e**.

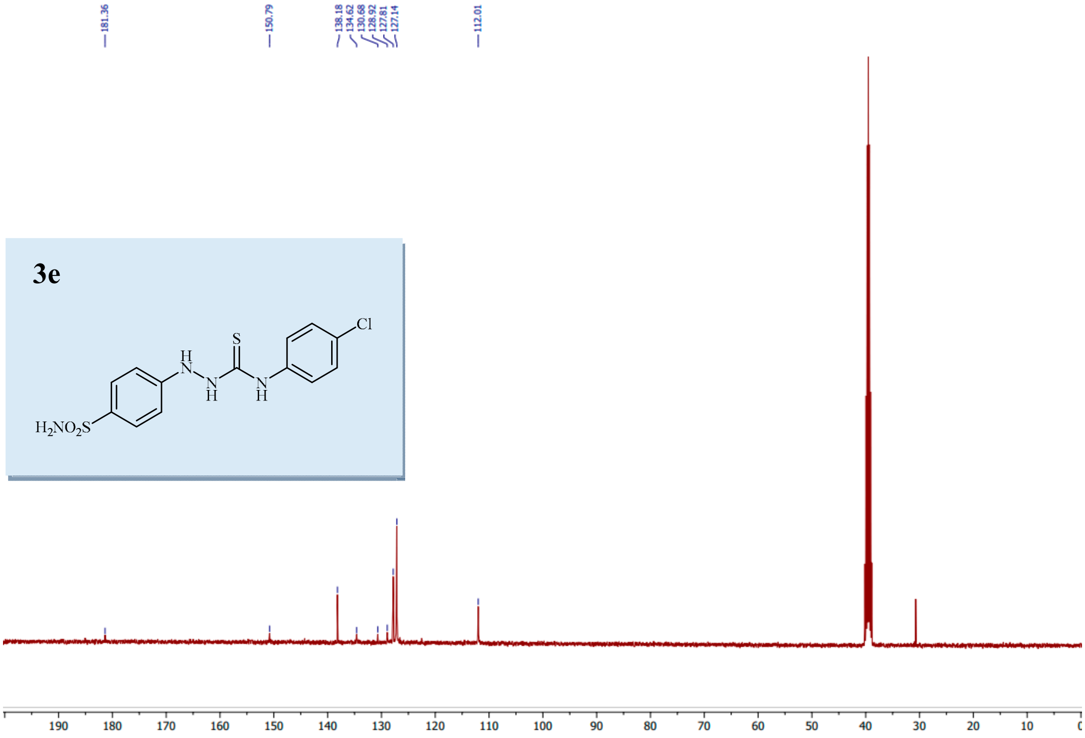

^{13}C NMR spectrum of compound **3e**.

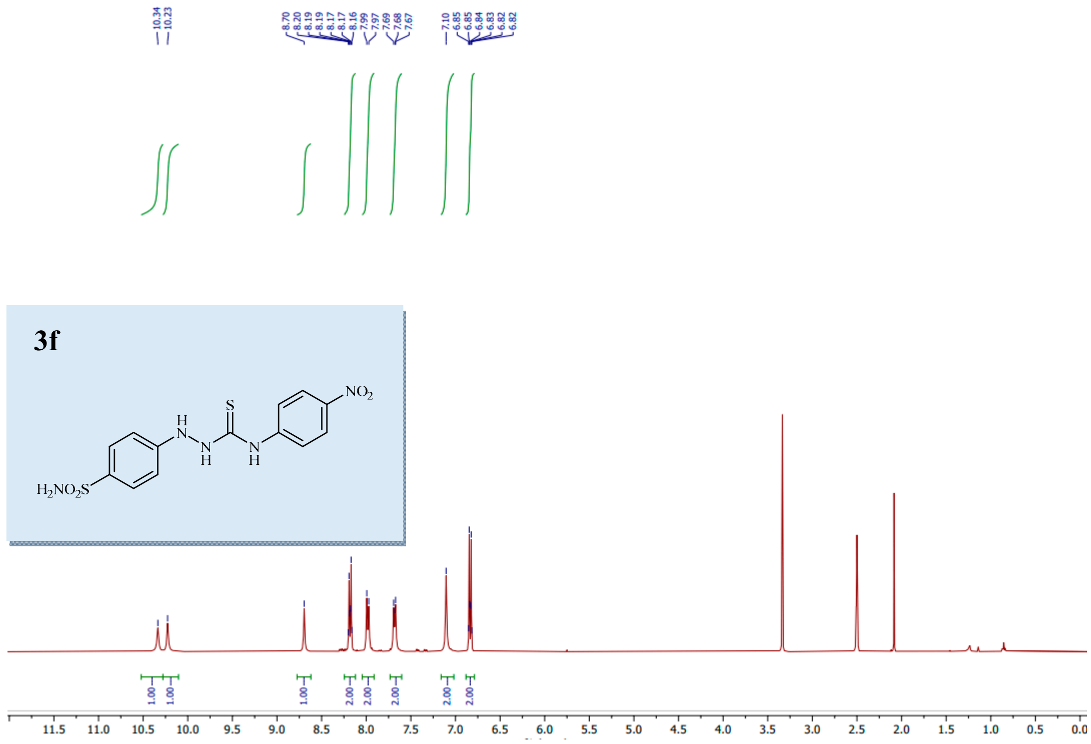

^{1}H NMR spectrum of compound **3f**.

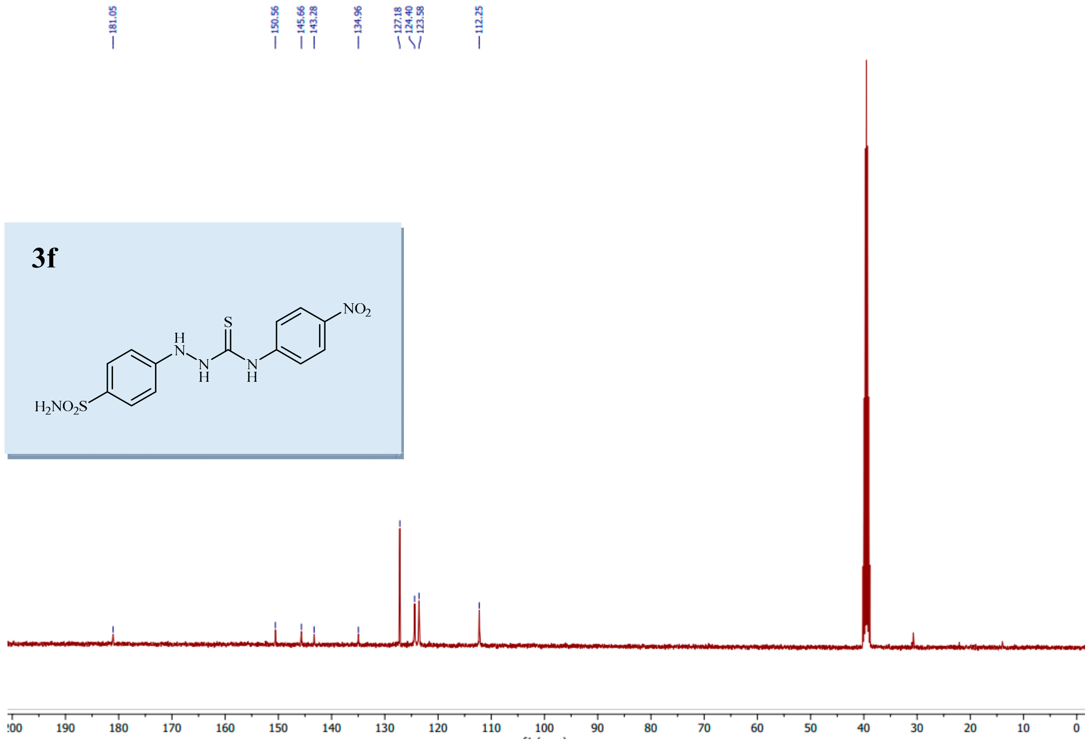

^{13}C NMR spectrum of compound **3f**.

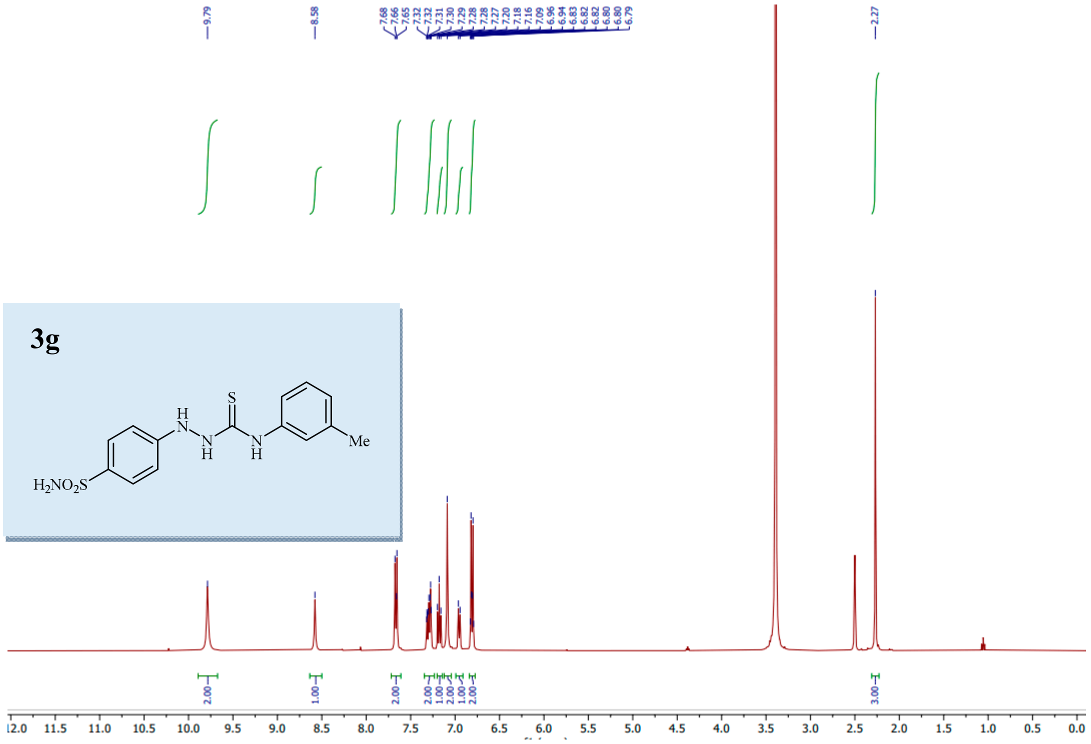

^{1}H NMR spectrum of compound **3g**.

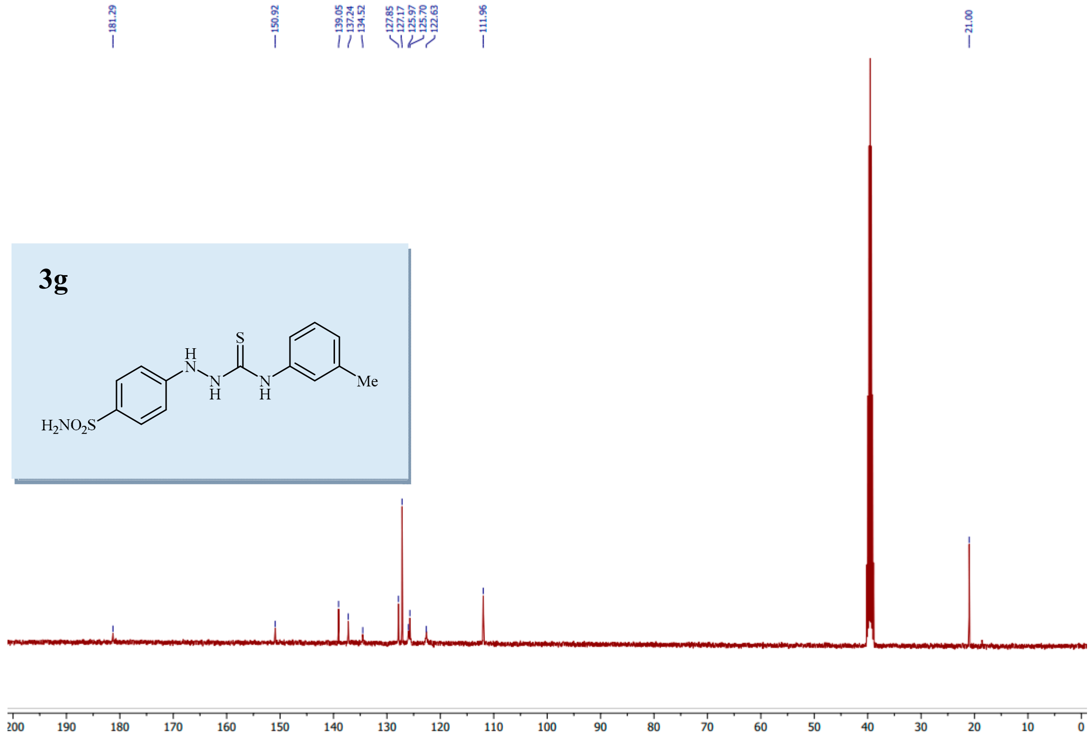

^{13}C NMR spectrum of compound **3g**.

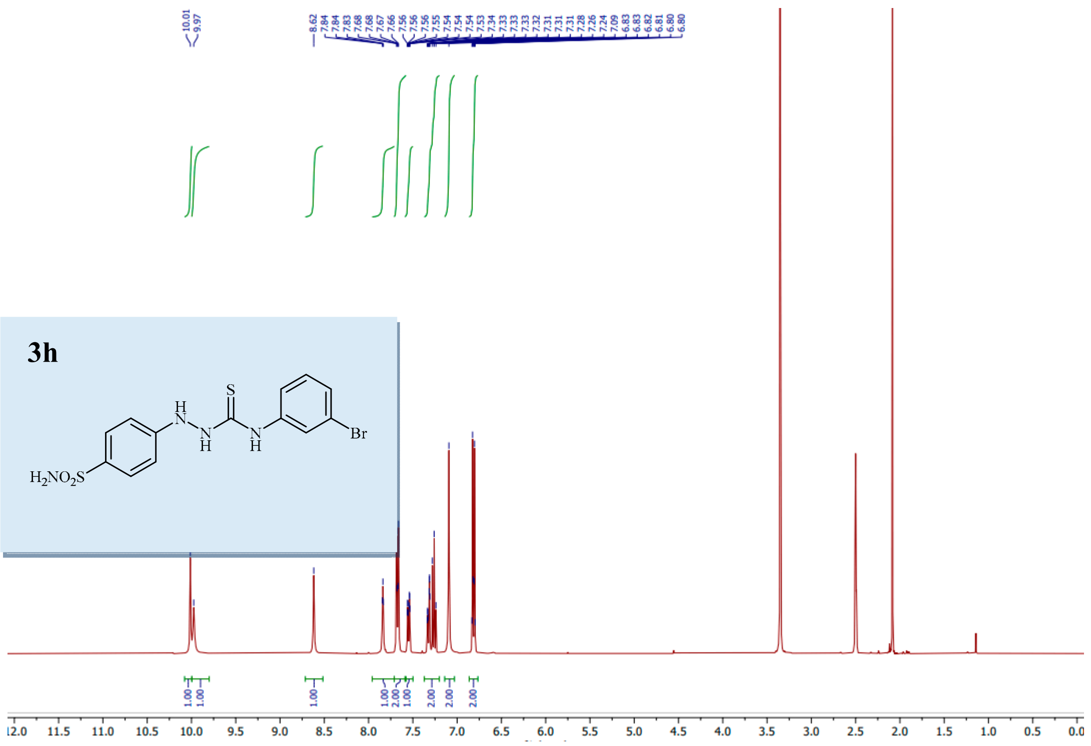

^{1}H NMR spectrum of compound **3h**.

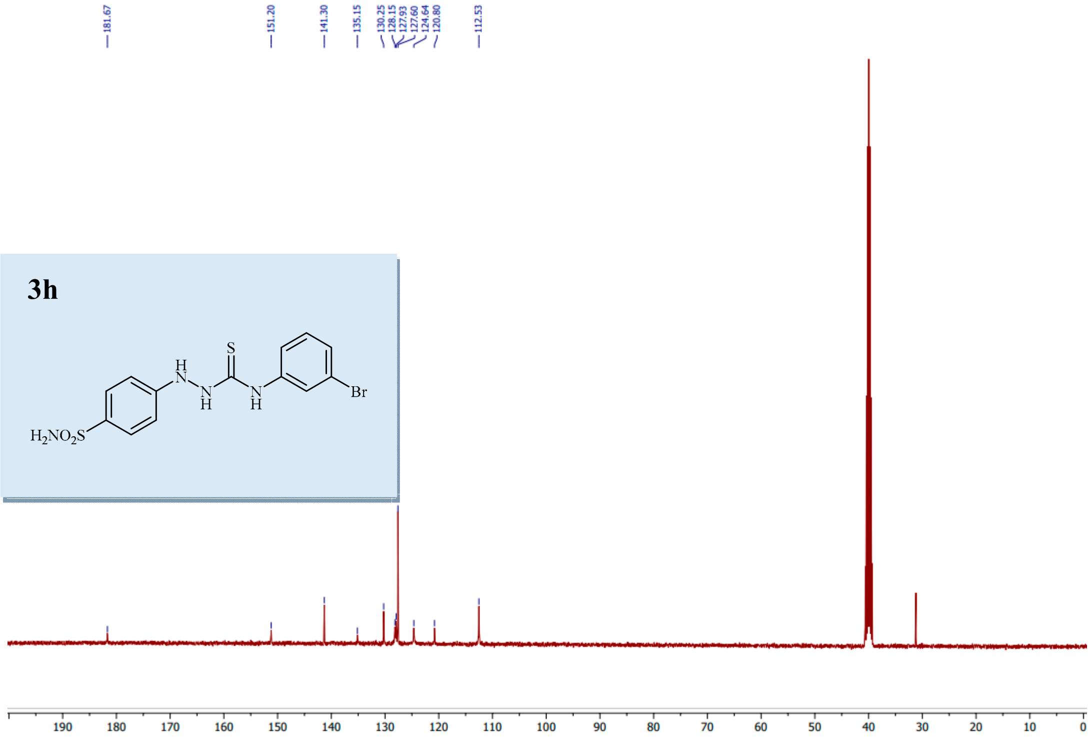

^{13}C NMR spectrum of compound **3h**.

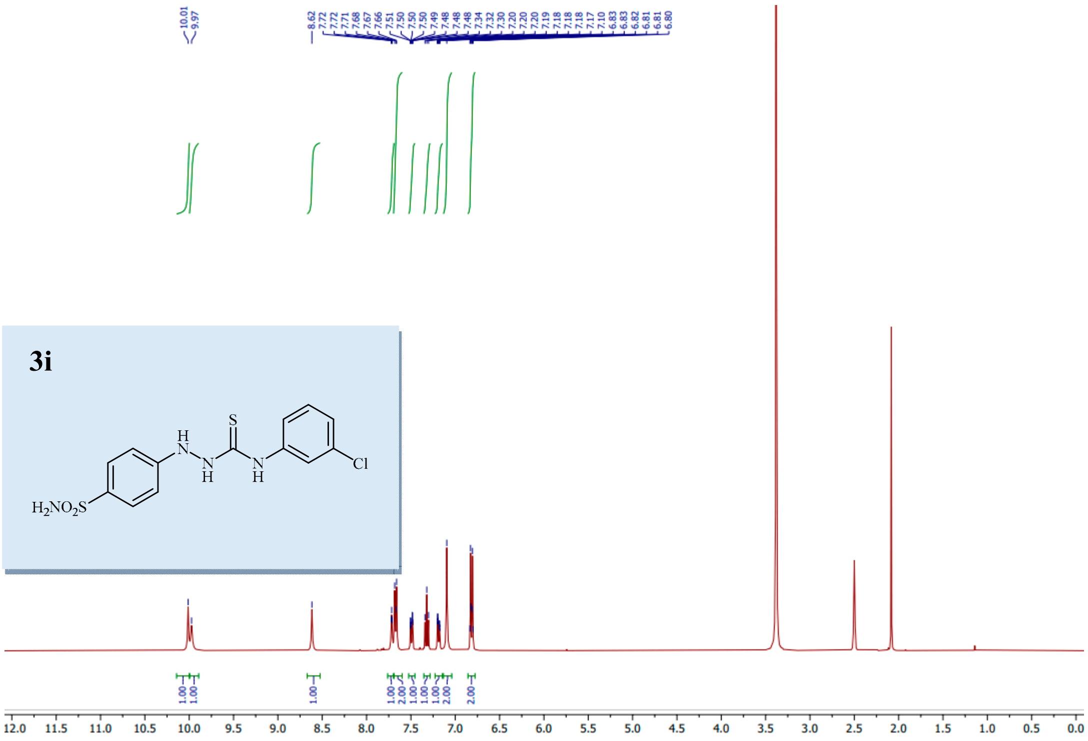

^{1}H NMR spectrum of compound **3i**.

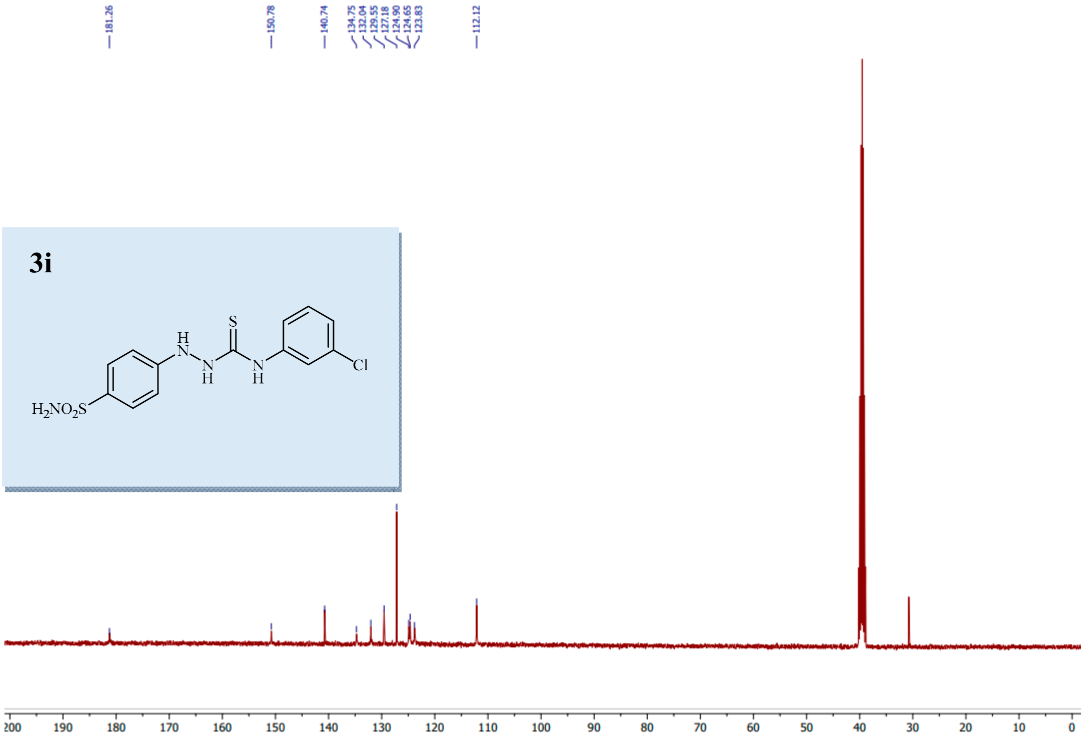

^{13}C NMR spectrum of compound **3i**.

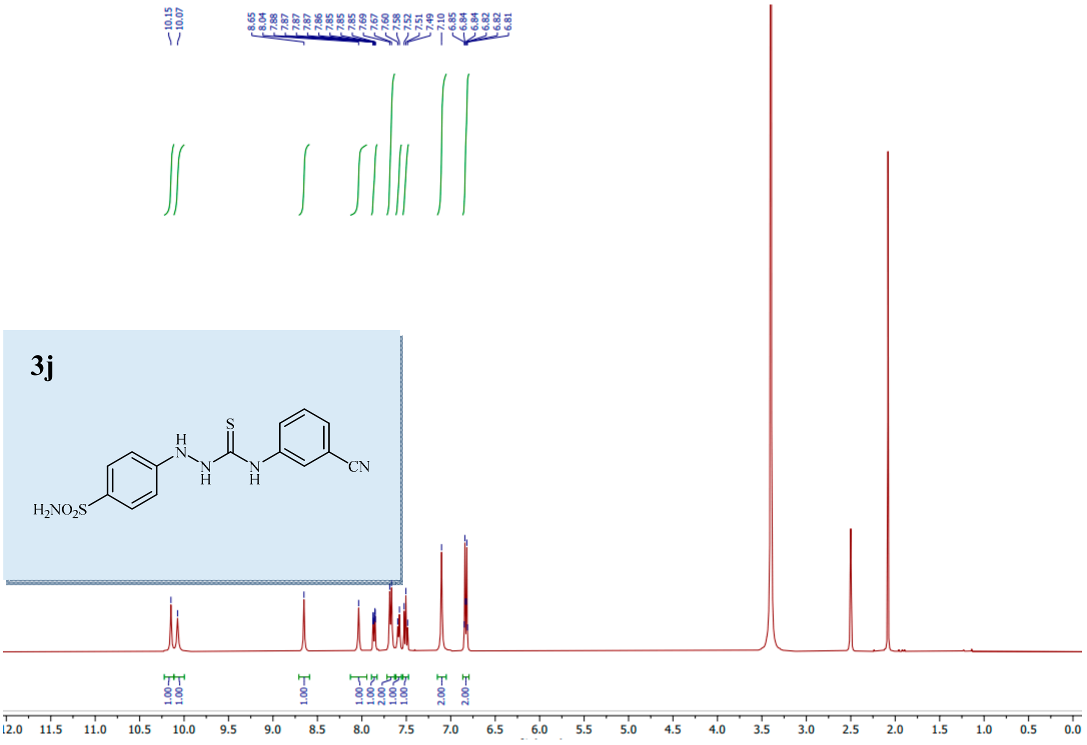

^{1}H NMR spectrum of compound **3j**.

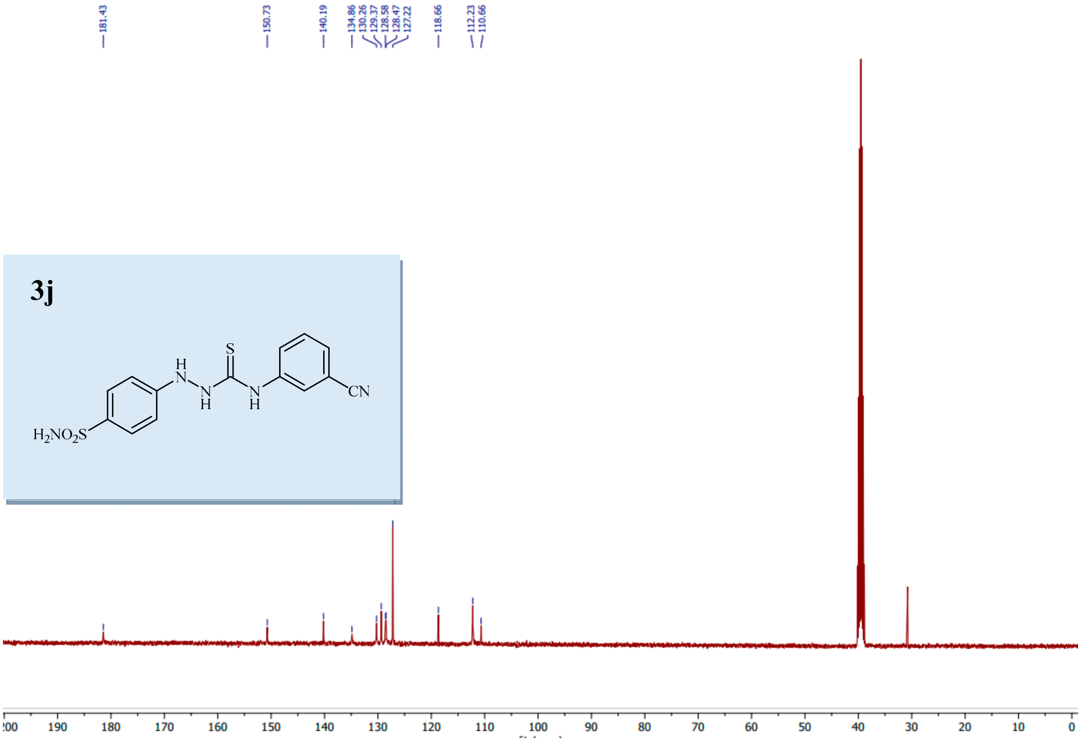

^{13}C NMR spectrum of compound **3j**.

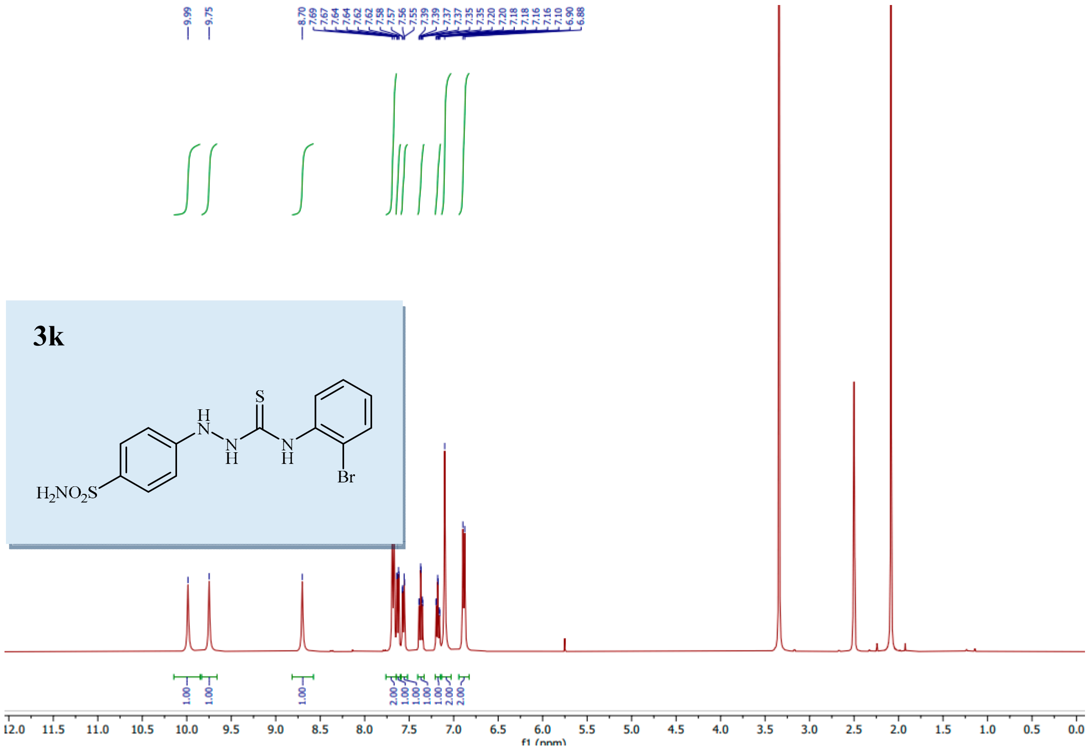

^{1}H NMR spectrum of compound **3k**.

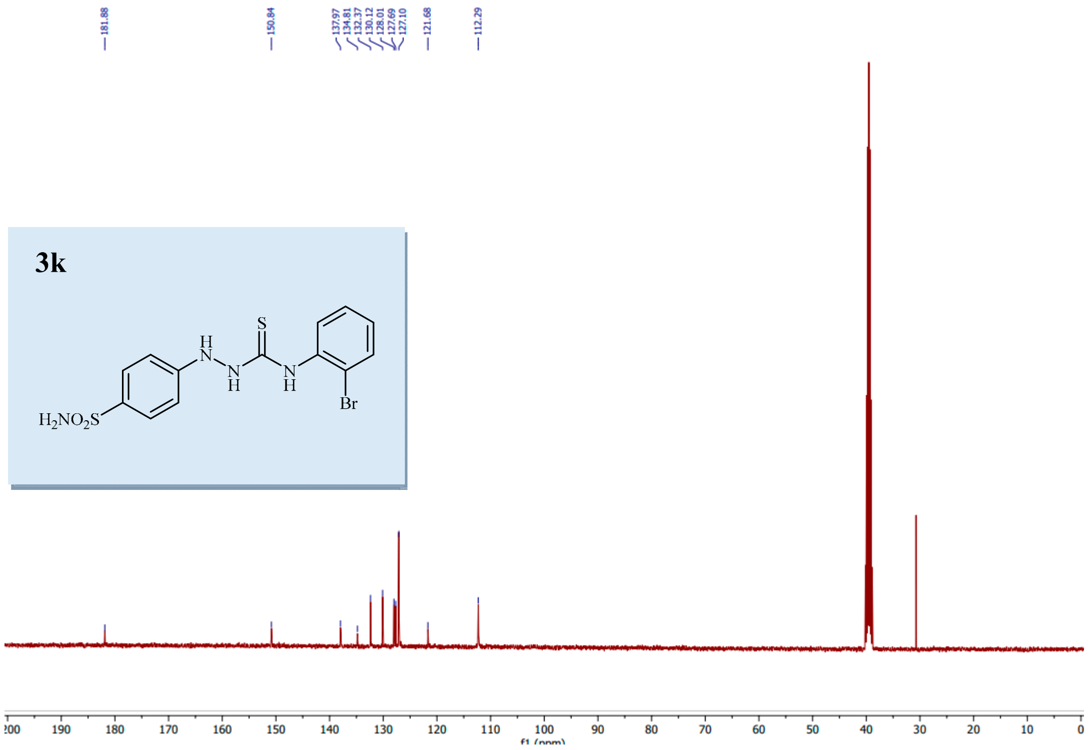

^{13}C NMR spectrum of compound **3k**.

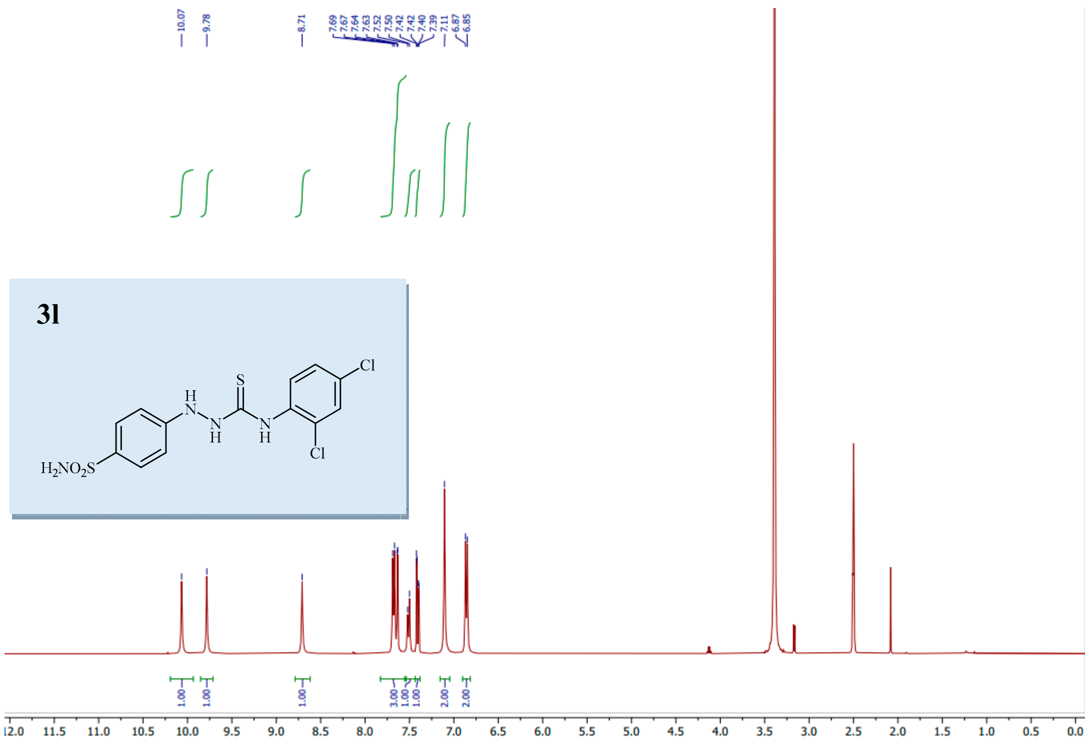

^{1}H NMR spectrum of compound **3l**.

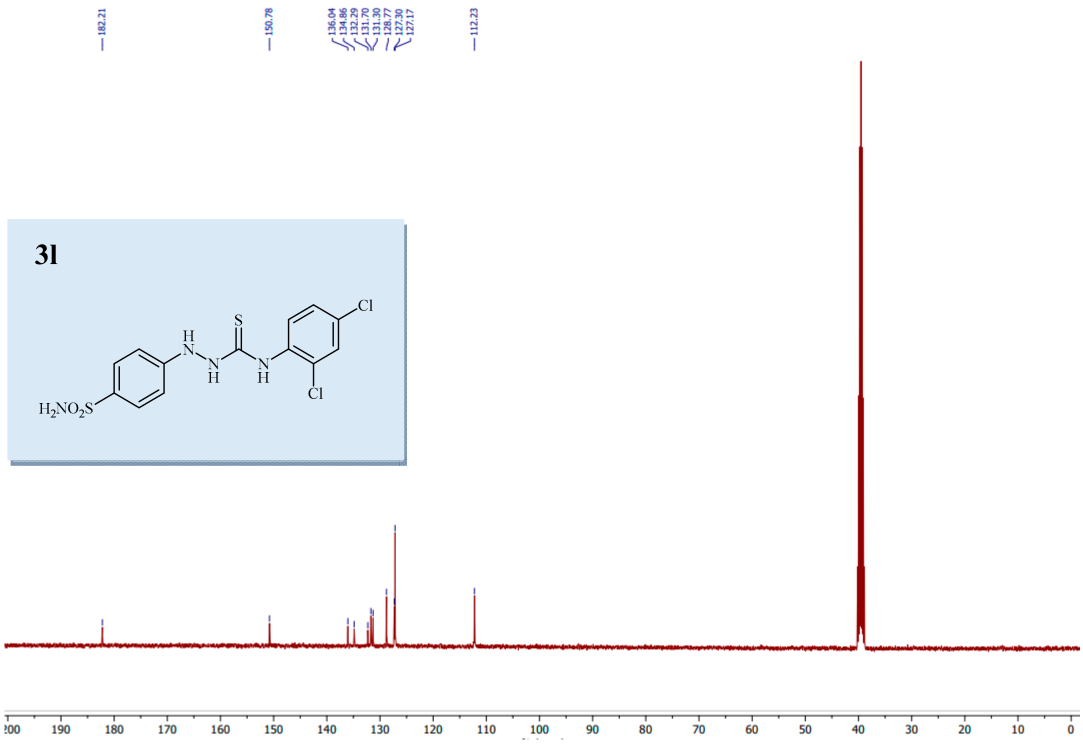

^{13}C NMR spectrum of compound **3l**.

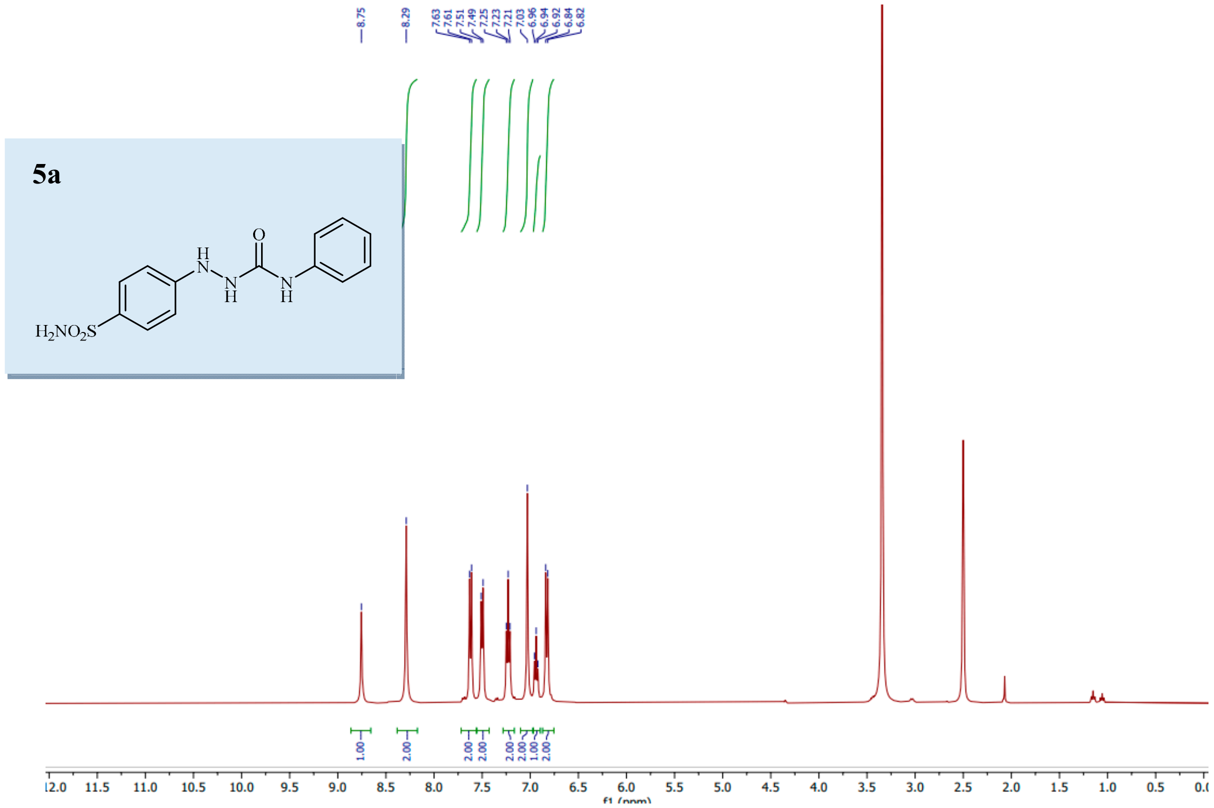

^{1}H NMR spectrum of compound **5a**.

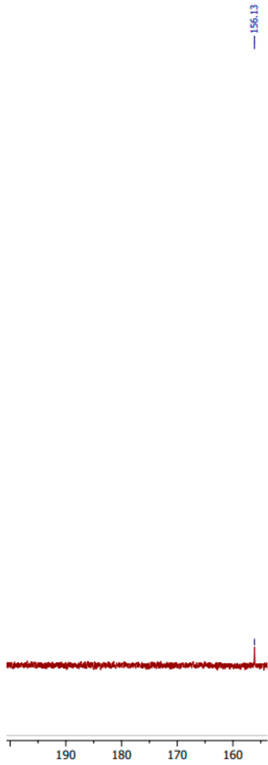

^{13}C NMR spectrum of compound **5a**.

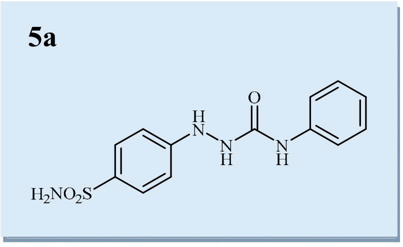

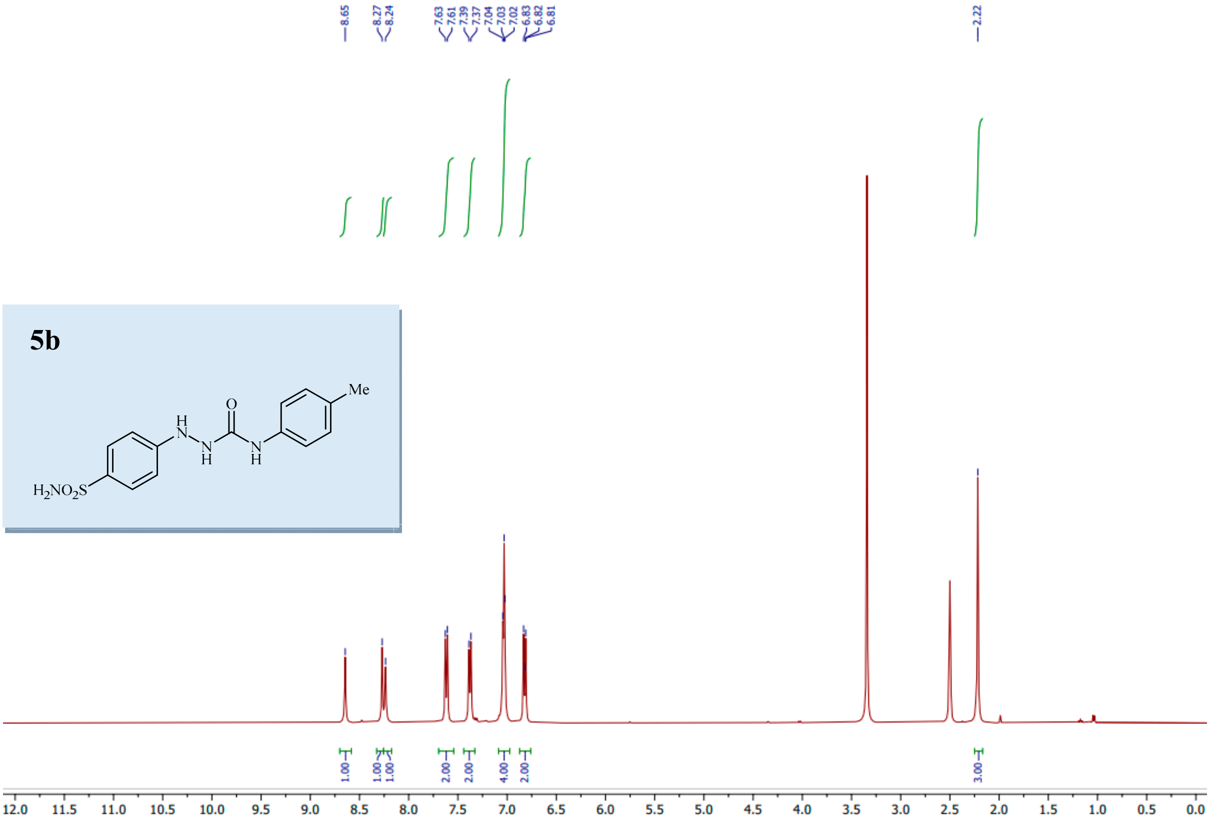

^{1}H NMR spectrum of compound **5b**.

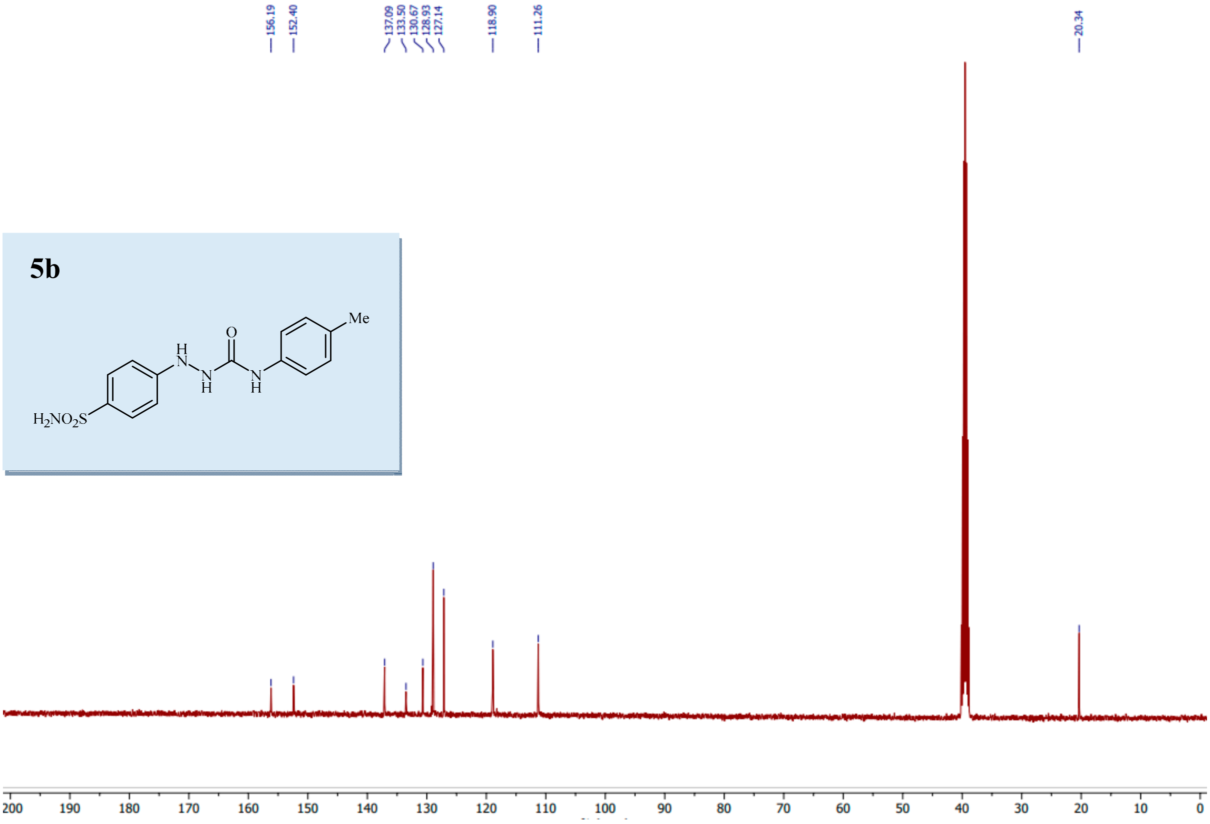

^{13}C NMR spectrum of compound **5b**.

Elemental Composition Report

Single Mass Analysis

Tolerance = 5.0 PPM / DBE: min = -0.5, max = 200.0
Element prediction: Off
Number of isotope peaks used for i-FIT = 5

Monoisotopic Mass, Even Electron Ions
348 formula(e) evaluated with 1 results within limits (all results (up to 1000) for each mass)
Elements Used:
C: 1-100 H: 1-150 N: 0-15 O: 0-15 S: 2-2

1842 Abdoli MA-2-110

OSI/FOKL-MS
Synapt G2-Si
1: TOF MS ES+
2.65e+006

HRMS_2024_10_533 593 (1.702) Cm (593:596-555:561)

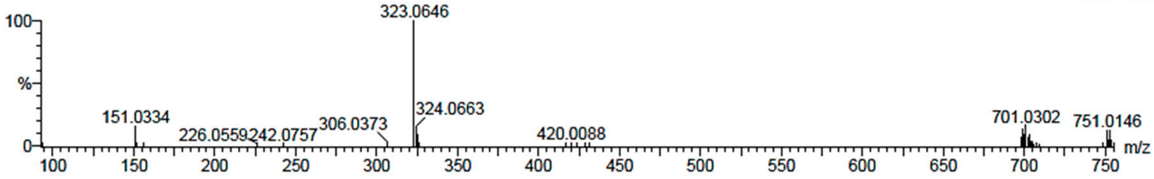

Minimum:				-0.5				
Maximum:		3.0	5.0	200.0				
Mass	Calc. Mass	mDa	PPM	DBE	i-FIT	Norm	Conf(%)	Formula
323.0646	323.0636	1.0	3.1	8.5	79.1	n/a	n/a	C13 H15 N4 O2 S2

HRMS spectra of compound **3a**

Elemental Composition Report

Single Mass Analysis

Tolerance = 3.0 PPM / DBE: min = -0.5, max = 200.0
Element prediction: Off
Number of isotope peaks used for i-FIT = 5

Monoisotopic Mass, Even Electron Ions
392 formula(e) evaluated with 1 results within limits (all results (up to 1000) for each mass)
Elements Used:
C: 1-50 H: 1-150 N: 0-15 O: 0-15 S: 2-2

1768 Abdoli MA-2-107

OSI/FOKL-MS
Synapt G2-Si
1: TOF MS ES+
2.46e+006

HRMS_2024_10_244 592 (1.700) Cm (591:597-566:575)

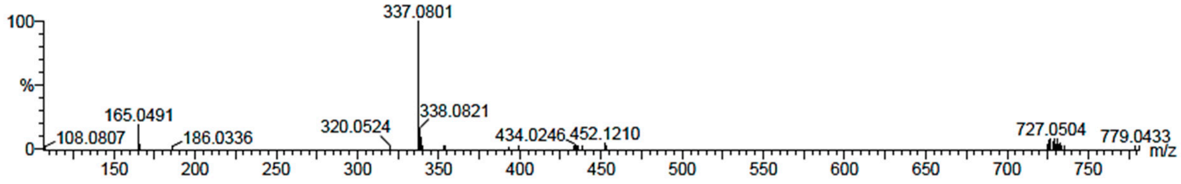

Minimum:				-0.5				
Maximum:		3.0	3.0	200.0				
Mass	Calc. Mass	mDa	PPM	DBE	i-FIT	Norm	Conf(%)	Formula
337.0801	337.0793	0.8	2.4	8.5	55.3	n/a	n/a	C14 H17 N4 O2 S2

HRMS spectra of compound **3b**

Elemental Composition Report

Page 1

Single Mass Analysis

Tolerance = 3.0 PPM / DBE: min = -0.5, max = 200.0
Element prediction: Off
Number of isotope peaks used for i-FIT = 5

Monoisotopic Mass, Even Electron Ions
484 formula(e) evaluated with 1 results within limits (all results (up to 1000) for each mass)
Elements Used:
C: 1-50 H: 1-150 N: 0-15 O: 0-15 S: 2-2

1780 Abdoli MA-2-123 — OSI/FOKL-MS
HRMS_2024_10_268 654 (1.874) Cm (650:654-634:641) — Synapt G2-Si
1: TOF MS ES+
3.07e+006

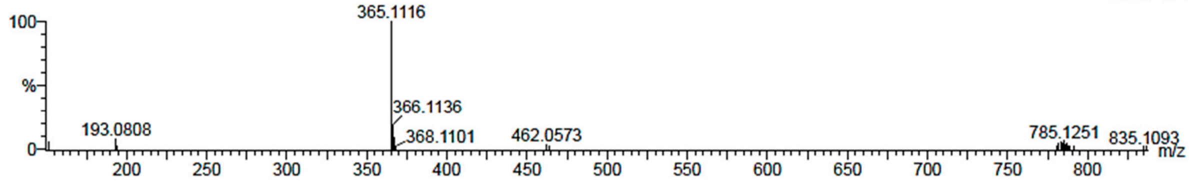

Minimum:				-0.5				
Maximum:		3.0	3.0	200.0				
Mass	Calc. Mass	mDa	PPM	DBE	i-FIT	Norm	Conf(%)	Formula
365.1116	365.1106	1.0	2.7	8.5	78.4	n/a	n/a	C16 H21 N4 O2 S2

HRMS spectra of compound **3c**

Elemental Composition Report

Page 1

Single Mass Analysis

Tolerance = 3.0 PPM / DBE: min = -0.5, max = 200.0
Element prediction: Off
Number of isotope peaks used for i-FIT = 5

Monoisotopic Mass, Even Electron Ions
323 formula(e) evaluated with 1 results within limits (all results (up to 1000) for each mass)
Elements Used:
C: 1-50 H: 1-150 N: 0-15 O: 0-15 S: 2-2 Br: 1-1

1771 Abdoli MA-2-113 — OSI/FOKL-MS
HRMS_2024_10_250 624 (1.787) Cm (623:627-598:605) — Synapt G2-Si
1: TOF MS ES+
3.47e+006

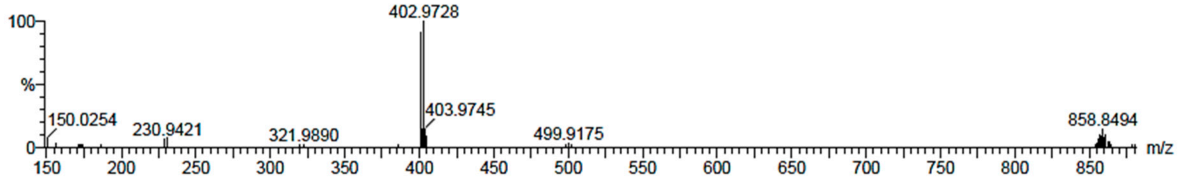

Minimum:				-0.5				
Maximum:		3.0	3.0	200.0				
Mass	Calc. Mass	mDa	PPM	DBE	i-FIT	Norm	Conf(%)	Formula
400.9748	400.9742	0.6	1.5	8.5	86.9	n/a	n/a	C13 H14 N4 O2 S2 Br

HRMS spectra of compound **3d**

Elemental Composition Report

Page 1

Single Mass Analysis

Tolerance = 3.0 PPM / DBE: min = -0.5, max = 200.0
Element prediction: Off
Number of isotope peaks used for i-FIT = 5

Monoisotopic Mass, Even Electron Ions
323 formula(e) evaluated with 1 results within limits (all results (up to 1000) for each mass)
Elements Used:
C: 1-50 H: 1-150 N: 0-15 O: 0-15 S: 2-2 Cl: 1-1

1772 Abdoli MA-2-114 — OSI/FOKL-MS Synapt G2-Si

HRMS_2024_10_252 613 (1.756) Cm (609:614-588:597) — 1: TOF MS ES+ 3.38e+006

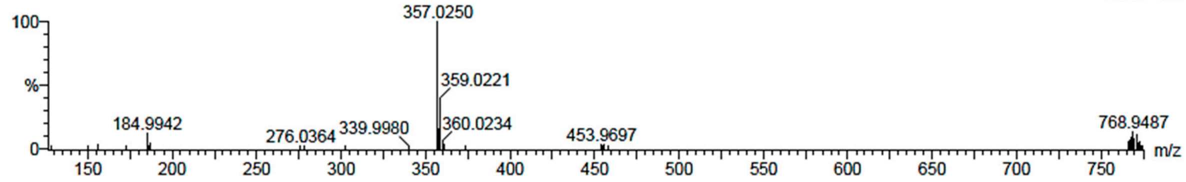

	Mass	Calc. Mass	mDa	PPM	DBE	i-FIT	Norm	Conf(%)	Formula
Minimum:					-0.5				
Maximum:			3.0	3.0	200.0				
	357.0250	357.0247	0.3	0.8	8.5	64.0	n/a	n/a	C13 H14 N4 O2 S2 Cl

HRMS spectra of compound **3e**

Elemental Composition Report

Page 1

Single Mass Analysis

Tolerance = 3.0 PPM / DBE: min = -0.5, max = 200.0
Element prediction: Off
Number of isotope peaks used for i-FIT = 5

Monoisotopic Mass, Even Electron Ions
479 formula(e) evaluated with 2 results within limits (all results (up to 1000) for each mass)
Elements Used:
C: 1-50 H: 1-150 N: 0-15 O: 0-15 S: 2-2

1776 Abdoli MA-2-118 — OSI/FOKL-MS Synapt G2-Si

HRMS_2024_10_260 593 (1.702) Cm (591:594-576:583) — 1: TOF MS ES+ 2.97e+006

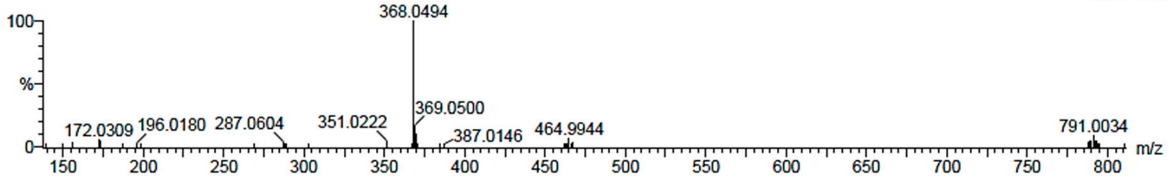

	Mass	Calc. Mass	mDa	PPM	DBE	i-FIT	Norm	Conf(%)	Formula
Minimum:					-0.5				
Maximum:			3.0	3.0	200.0				
	368.0494	368.0487	0.7	1.9	9.5	82.7	0.976	37.67	C13 H14 N5 O4 S2
		368.0501	-0.7	-1.9	14.5	82.2	0.473	62.33	C14 H10 N9 S2

HRMS spectra of compound **3f**

Elemental Composition Report

Page 1

Single Mass Analysis

Tolerance = 3.0 PPM / DBE: min = -0.5, max = 200.0
Element prediction: Off
Number of isotope peaks used for i-FIT = 5

Monoisotopic Mass, Even Electron Ions
392 formula(e) evaluated with 1 results within limits (all results (up to 1000) for each mass)
Elements Used:
C: 1-50 H: 1-150 N: 0-15 O: 0-15 S: 2-2

1779 Abdoli MA-2-122

HRMS_2024_10_266 591 (1.696) Cm (589:591-572:580)

OSI/FOKL-MS
Synapt G2-Si
1: TOF MS ES+
2.07e+006

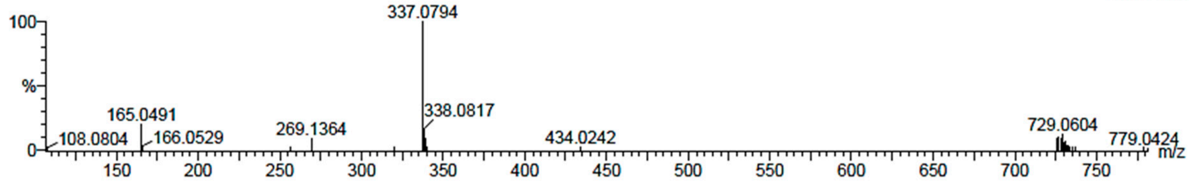

Minimum:				-0.5				
Maximum:		3.0	3.0	200.0				
Mass	Calc. Mass	mDa	PPM	DBE	i-FIT	Norm	Conf(%)	Formula
337.0794	337.0793	0.1	0.3	8.5	74.3	n/a	n/a	C14 H17 N4 O2 S2

HRMS spectra of compound **3g**

Elemental Composition Report

Page 1

Single Mass Analysis

Tolerance = 3.0 PPM / DBE: min = -0.5, max = 200.0
Element prediction: Off
Number of isotope peaks used for i-FIT = 5

Monoisotopic Mass, Even Electron Ions
323 formula(e) evaluated with 1 results within limits (all results (up to 1000) for each mass)
Elements Used:
C: 1-50 H: 1-150 N: 0-15 O: 0-15 S: 2-2 Br: 1-1

1773 Abdoli MA-2-115

HRMS_2024_10_254 622 (1.781) Cm (620:625-597:601)

OSI/FOKL-MS
Synapt G2-Si
1: TOF MS ES+
1.69e+006

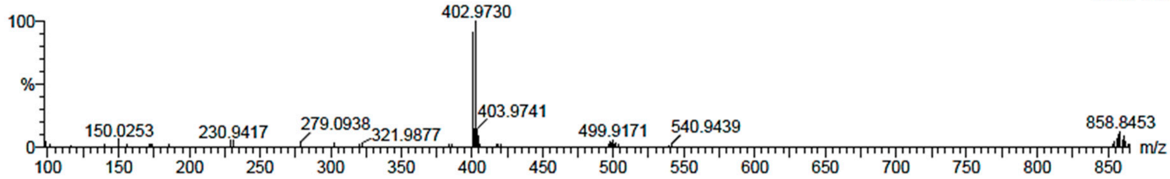

Minimum:				-0.5				
Maximum:		3.0	3.0	200.0				
Mass	Calc. Mass	mDa	PPM	DBE	i-FIT	Norm	Conf(%)	Formula
400.9746	400.9742	0.4	1.0	8.5	42.0	n/a	n/a	C13 H14 N4 O2 S2 Br

HRMS spectra of compound **3g**

Elemental Composition Report

Page 1

Single Mass Analysis

Tolerance = 3.0 PPM / DBE: min = -0.5, max = 200.0
Element prediction: Off
Number of isotope peaks used for i-FIT = 5

Monoisotopic Mass, Even Electron Ions
323 formula(e) evaluated with 1 results within limits (all results (up to 1000) for each mass)
Elements Used:
C: 1-50 H: 1-150 N: 0-15 O: 0-15 S: 2-2 Cl: 1-1

1775 Abdoli MA-2-117

OSI/FOKL-MS
Synapt G2-Si
1: TOF MS ES+
6.42e+006

HRMS_2024_10_258 615 (1.762) Cm (610:615-593:600)

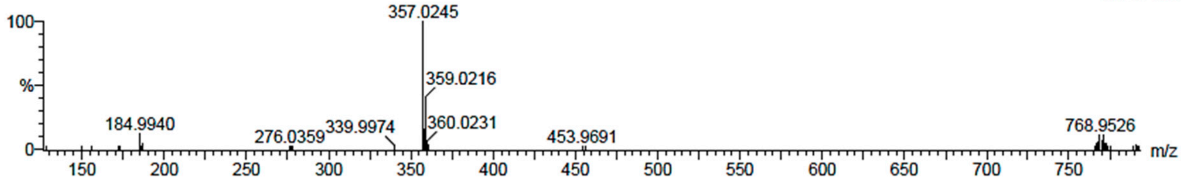

Minimum:				-0.5				
Maximum:		3.0	3.0	200.0				

Mass	Calc. Mass	mDa	PPM	DBE	i-FIT	Norm	Conf(%)	Formula
357.0245	357.0247	-0.2	-0.6	8.5	91.5	n/a	n/a	C13 H14 N4 O2 S2 Cl

HRMS spectra of compound **3i**

Elemental Composition Report

Page 1

Single Mass Analysis

Tolerance = 3.0 PPM / DBE: min = -0.5, max = 200.0
Element prediction: Off
Number of isotope peaks used for i-FIT = 5

Monoisotopic Mass, Even Electron Ions
415 formula(e) evaluated with 1 results within limits (all results (up to 1000) for each mass)
Elements Used:
C: 1-50 H: 1-150 N: 0-15 O: 0-15 S: 2-2

1778 Abdoli MA-2-120

OSI/FOKL-MS
Synapt G2-Si
1: TOF MS ES+
1.76e+006

HRMS_2024_10_264 563 (1.614) Cm (562:567-544:549)

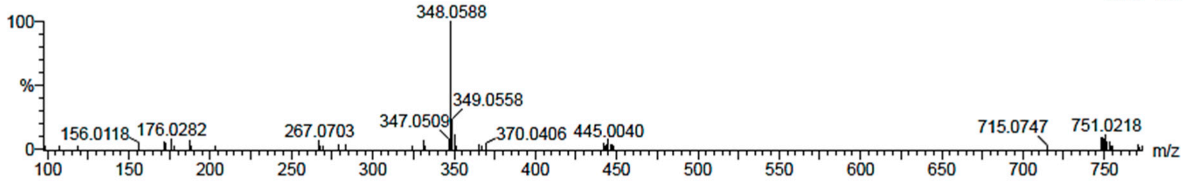

Minimum:				-0.5				
Maximum:		3.0	3.0	200.0				

Mass	Calc. Mass	mDa	PPM	DBE	i-FIT	Norm	Conf(%)	Formula
348.0588	348.0589	-0.1	-0.3	10.5	64.6	n/a	n/a	C14 H14 N5 O2 S2

HRMS spectra of compound **3j**

Elemental Composition Report

Single Mass Analysis
Tolerance = 3.0 PPM / DBE: min = -0.5, max = 200.0
Element prediction: Off
Number of isotope peaks used for i-FIT = 5

Monoisotopic Mass, Even Electron Ions
323 formula(e) evaluated with 1 results within limits (all results (up to 1000) for each mass)
Elements Used:
C: 1-50 H: 1-150 N: 0-15 O: 0-15 S: 2-2 Br: 1-1

1774 Abdoli MA-2-116 OSI/FOKL-MS
Synapt G2-Si
HRMS_2024_10_256 587 (1.680) Cm (585:590-567:572) 1: TOF MS ES+
2.76e+006

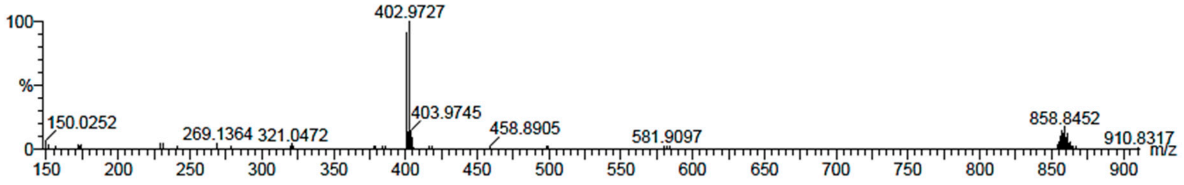

Mass	Calc. Mass	mDa	PPM	DBE	i-FIT	Norm	Conf(%)	Formula
Minimum:				-0.5				
Maximum:		3.0	3.0	200.0				
400.9747	400.9742	0.5	1.2	8.5	42.6	n/a	n/a	C13 H14 N4 O2 S2 Br

HRMS spectra of compound **3k**

Elemental Composition Report

Single Mass Analysis
Tolerance = 3.0 PPM / DBE: min = -0.5, max = 200.0
Element prediction: Off
Number of isotope peaks used for i-FIT = 5

Monoisotopic Mass, Even Electron Ions
323 formula(e) evaluated with 1 results within limits (all results (up to 1000) for each mass)
Elements Used:
C: 1-50 H: 1-150 N: 0-15 O: 0-15 S: 2-2 Cl: 2-2

1777 Abdoli MA-2-119 OSI/FOKL-MS
Synapt G2-Si
HRMS_2024_10_262 637 (1.822) Cm (632:639-606:617) 1: TOF MS ES+
1.48e+006

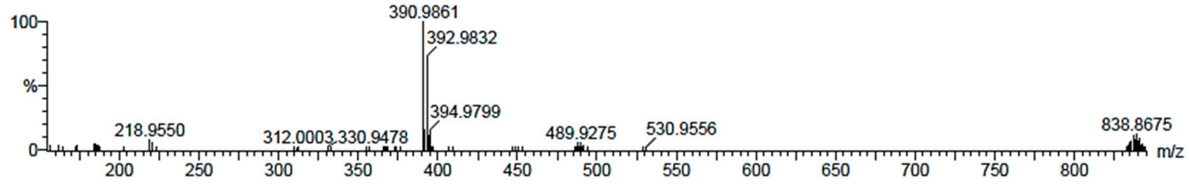

Mass	Calc. Mass	mDa	PPM	DBE	i-FIT	Norm	Conf(%)	Formula
Minimum:				-0.5				
Maximum:		3.0	3.0	200.0				
390.9861	390.9857	0.4	1.0	8.5	37.2	n/a	n/a	C13 H13 N4 O2 S2 Cl2

HRMS spectra of compound **3l**

Elemental Composition Report **Page 1**

Single Mass Analysis
Tolerance = 3.0 PPM / DBE: min = -0.5, max = 200.0
Element prediction: Off
Number of isotope peaks used for i-FIT = 5

Monoisotopic Mass, Even Electron Ions
399 formula(e) evaluated with 1 results within limits (all results (up to 1000) for each mass)
Elements Used:
C: 1-50 H: 1-150 N: 0-15 O: 0-15 S: 1-1
1767 Abdoli MA-2-106

OSI/FOKL-MS
Synapt G2-Si
1: TOF MS ES+
1.65e+006

HRMS_2024_10_242 531 (1.521) Cm (531:533-509:517)

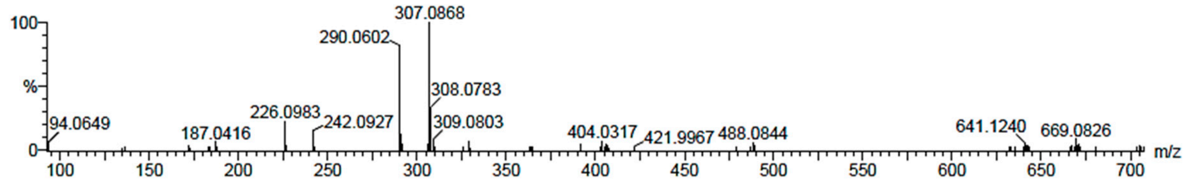

Minimum:				-0.5				
Maximum:		3.0	3.0	200.0				
Mass	Calc. Mass	mDa	PPM	DBE	i-FIT	Norm	Conf(%)	Formula
307.0868	307.0865	0.3	1.0	8.5	64.9	n/a	n/a	C13 H15 N4 O3 S

HRMS spectra of compound **5a**

Elemental Composition Report **Page 1**

Single Mass Analysis
Tolerance = 3.0 PPM / DBE: min = -0.5, max = 200.0
Element prediction: Off
Number of isotope peaks used for i-FIT = 5

Monoisotopic Mass, Even Electron Ions
445 formula(e) evaluated with 1 results within limits (all results (up to 1000) for each mass)
Elements Used:
C: 1-50 H: 1-150 N: 0-15 O: 0-15 S: 1-1
1769 Abdoli MA-2-108

OSI/FOKL-MS
Synapt G2-Si
1: TOF MS ES+
3.07e+006

HRMS_2024_10_246 577 (1.652) Cm (577:583-544:552)

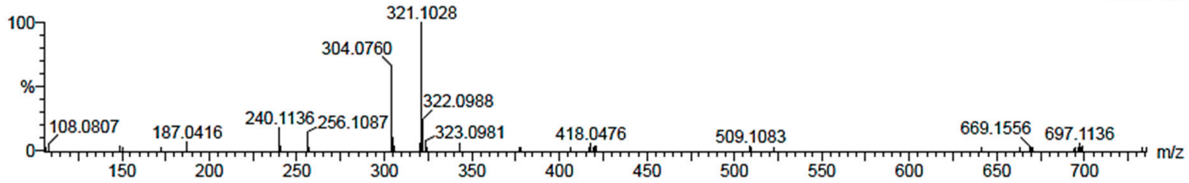

Minimum:				-0.5				
Maximum:		3.0	3.0	200.0				
Mass	Calc. Mass	mDa	PPM	DBE	i-FIT	Norm	Conf(%)	Formula
321.1028	321.1021	0.7	2.2	8.5	86.8	n/a	n/a	C14 H17 N4 O3 S

HRMS spectra of compound **5b**
